# Integrated Physiological and Transcriptomic Analyses Reveal Secondary Metabolic Responses of *Prunella vulgaris* Seedlings to Sodium Nitroprusside Treatment

**DOI:** 10.3390/ijms27219585

**Published:** 2026-10-27

**Authors:** Zhiguo Gong, Muyun Zhu, Qian Zuo, Xi Zou, Xiangling Hu, Yufan Li, Wuzhimo Ergu, Rong Deng, Tao Chen, Bingcong Xing, Zaibiao Zhu, Liping Cao, Yuhang Chen

**Affiliations:** 1 College of Pharmacy, Chengdu Medical College, Chengdu 610500, China; 2 Key Laboratory of Structure-Specific Small Molecule Drugs at Chengdu Medical College of Sichuan Province, School of Pharmacy, Chengdu Medical College, Chengdu 610500, China; 3 State Key Laboratory for Development and Utilization of Forest Food Resources, Zhejiang A&F University, Hangzhou 311300, China; 4 Institute of Chinese Medicinal Materials, Nanjing Agricultural University, Nanjing 210095, China

**Keywords:** physiological and biochemical, *Prunella vulgaris* L., secondary metabolism, sodium nitroprusside, transcriptomic

## Abstract

*Prunella vulgaris* L. is a valuable edible and medicinal industrial crop widely cultivated and used in Asia and Europe. Although exogenous sodium nitroprusside (SNP, a NO donor) is known to increase the biosynthesis of bioactive polyphenols and triterpenoids in this plant, its precise regulatory network remains unclear. Here, we integrated physiological and transcriptomic analyses to decipher how exogenous sodium nitroprusside (SNP, 100 μM) reprograms secondary metabolism in *P. vulgaris* seedling leaves at 2, 24, and 48 h post treatment. The results indicated that SNP treatment significantly altered antioxidant and redox responses, elevated osmotic regulators, and increased accumulation of key bioactive compounds (such as rosmarinic acid and ursolic acid), accompanied by increased in vitro antioxidant activity. At 24 h post treatment, our comparative analysis revealed 4341 differentially expressed transcripts (DETs). Exogenous SNP treatment is associated with phytohormone signal transduction, Ca^2+^ signaling, and secondary metabolite biosynthesis. SNP treatment markedly increased endogenous jasmonic acid (JA) levels and upregulated the expression of core transcription factors (MYBs, bHLHs, WRKYs, and NACs), along with increased expression of JA biosynthesis transcripts (e.g., *PvLOXs*, *PvAOC*, and *PvACX*), as well as key transcripts involved in polyphenol biosynthesis (e.g., *PvPAL*, *PvTAT*, and *PvRAS*) and triterpenoid biosynthesis (e.g., *PvHMGS*, *PvDXS*, and *PvSQS*). Our findings provide a systematic view of the physiological, hormonal, and transcriptional responses through which SNP is associated with changes in secondary metabolism in *P. vulgaris* and provide a theoretical basis for future studies on foliar SNP application to improve the pharmaceutical quality.

## 1. Introduction

*Prunella vulgaris* L. (Lamiaceae) is an important medicinal and edible plant widely utilized in Asia and Europe for treating conditions such as thyroid gland malfunctions, infectious hepatitis, and mastitis [1]. This species is broadly distributed across temperate and subtropical regions of the Northern Hemisphere, including East Asia, South Asia, West Asia, Europe, North Africa, and North America [2]. In China, *P. vulgaris* is predominantly found in the Yangtze River Basin, where it typically inhabits grassy slopes, shrublands, and forest edges at altitudes ranging from 450 to 3000 m, reflecting its inherent adaptability to low-light understory environments [3]. Historically, the seedling leaves of *P. vulgaris* have been consumed as a seasonal vegetable in China since the Ming Dynasty, a nearly 400-year history of human consumption [4,5]. In addition to its culinary use, the plant serves as a key raw material for functional herbal teas with hepatoprotective effects and is cultivated as an ornamental plant in southern China [6,7]. In Europe, it is mixed with honey as a tonic, while its extracts are employed as natural preservatives in the food industry [8]. Owing to its versatile applications in medicine, food, and horticulture, national forestry statistics indicate that the annual market demand for *P. vulgaris* in China is approximately 5000–10,000 tons [9]. Consequently, artificial cultivation has become the main method for mitigating the imbalance between supply and demand.

In recent years, understory ecological cultivation has become a key model for traditional Chinese medicine (TCM) agriculture, which aligns with China’s national ecological strategy and rural revitalization policies [10]. Taking advantage of its natural adaptability to low-light understory habitats, the scale of artificial understory cultivation of *P. vulgaris* is gradually expanding in the hilly forest areas of Southwest China, supported by national policies for TCM ecological agriculture [11]. Previous studies have shown that moderate shading (30–80% of full sunlight) can significantly increase the yield and active ingredient content of various medicinal herbs, such as *Panax ginseng* [12] and *Atractylodes lancea* (Thunb.) DC. [13], *Epimedium pubescens* Maxim. [14], and *Salvia miltiorrhiza* Bunge [15], leading to the establishment of standardized technical protocols for this cultivation method. Our prior research revealed that various abiotic stresses, including UV-B radiation, UV solar exclusion, drought, and soil nutrient shortage, can facilitate the accumulation of polyphenols and triterpenoids in *P. vulgaris*. This increase is mediated through signaling molecules such as hydrogen peroxide (H_2_O_2_) and endogenous phytohormones, including salicylic acid (SA) and methyl jasmonate (MeJA) [2,6,16,17,18]. Preliminary observations from our group further revealed that compared with that under full sunlight, endogenous nitric oxide (NO) levels and rosmarinic acid (RA) content in *P. vulgaris* spikes are significantly higher under moderately weak light conditions (25–75% shading); for example, in our preliminary experiment, compared with a full sunlight control, 75% shading increased the leaf NO content by approximately 20.1%, with the RA and ursolic acid (UA) contents increasing significantly by 138.9% and 15.6%, respectively (our unpublished data). Therefore, sodium nitroprusside (SNP), as an exogenous NO donor, was used in this study to investigate its potential effects on the accumulation of active components. Although NO is recognized as a crucial stress signaling molecule, the specific molecular cascade network underlying the regulation of secondary metabolism induced by exogenous SNP treatment in *P. vulgaris*, particularly its potential interplay with hormone pathways (e.g., JA, SA, and IAA) and key biosynthetic transcripts (e.g., *PvRAS*, *PvTAT*, and *PvDXS*), remain largely unclear. This knowledge gap severely restricts the targeted optimization and large-scale application of understory cultivation techniques for this valuable species. Therefore, this study aims to investigate the physiological and transcriptional responses of *P. vulgaris* seedlings to exogenous SNP treatment, providing a theoretical basis for future field studies on understory ecological cultivation.

Polyphenols and triterpenoids are the main bioactive components that accumulate in *P. vulgaris*, and these compounds are well known for their antioxidant, antitumor, and anti-inflammatory activities [7,19]. The contents of specific polyphenolics such as RA and triterpenoids, including UA, are key indicators for assessing the quality of *P. vulgaris* in the Chinese Pharmacopoeia [20,21]. SNP is a commonly used NO donor that releases NO via reductive decomposition [22]. Previous studies have shown that exogenous SNP treatment not only regulates plant growth, development, and tolerance to abiotic stress [23,24] but also increases antioxidant enzyme activity and stimulates the accumulation of secondary metabolites in various medicinal plant species [25,26,27]. As a small, diffusible free radical, NO has been demonstrated to induce flavonoid synthesis in the roots and rhizomes of *Saposhnikovia divaricata* [27], promote phenolic acid accumulation in the leaves of *Salvia officinalis* and hairy roots of *S. miltiorrhiza* [28,29], and stimulate terpenoid production in the leaves of *Artemisia annua* [30]. Thus, elucidating the physiological and transcriptomic responses of *P. vulgaris* to SNP treatment, which may involve NO signaling, is highly important.

Transcriptomic sequencing technologies have been extensively applied to clarify the molecular mechanisms underlying the biosynthesis of bioactive components in *P. vulgaris*. For instance, our previous combined physiological and transcriptomic study demonstrated that changes in polyphenol and triterpenoid biosynthesis are crucial for plant adaptation to H_2_O_2_ stress [16]. Similarly, integrated transcriptomic, metabolomic, and proteomic analyses have revealed that phenylpropanoid and triterpenoid biosynthesis pathways are essential for the growth and development of *P. vulgaris* spikes, revealing that RA accumulation prior to spica ripening largely depends on the upregulated expression of key pathway enzyme-coding transcripts [8]. Furthermore, the recent assembly of a chromosome-level genome of *P. vulgaris* has provided a foundational resource, facilitating the preliminary elucidation of the biosynthetic pathways for its pentacyclic triterpenoids (e.g., UA and oleanolic acid) [9,31]. Using this high-quality genome sequence, we identified 51 candidate enzyme-encoding transcripts involved in RA biosynthesis and functionally characterized key downstream acyltransferases, confirming that *PvRAS3* is the principal enzyme that catalyzes the condensation step, with *PvRAS4* playing a subsidiary role [1]. Although these transcriptome-based studies have successfully delineated RA and UA biosynthesis pathways under specific stressors or at defined developmental stages, the physiological responses and transcriptomic networks in response to SNP treatment in *P. vulgaris* seedling leaves remain unexplored. Therefore, an integrated physiological and transcriptomic approach is essential for exploring the physiological and transcriptional responses associated with SNP treatment [32].

Preliminary experiments (Supplementary Figure S1) revealed that foliar application of 100 μM SNP was more effective than other concentrations (0, 50, and 200 μM) at promoting the accumulation of key bioactive metabolites (RA and UA) in *P. vulgaris* leaves. Additionally, the contents of UA and total flavonoids were highest at 24 h among the time points examined but then decreased at 48 and 72 h. Therefore, 100 μM SNP and the 2, 24, and 48 h time points were selected for further analysis. The primary purpose of this study was to investigate how exogenous 100 μM SNP affects the biosynthesis of polyphenols and triterpenoids in *P. vulgaris* seedlings. We comprehensively analyzed the antioxidant defense system, osmotic regulators, endogenous phytohormones, secondary metabolites, and antioxidant activity of ethanol extracts. At 24 h, we identified differentially expressed transcripts (DETs) involved in the biosynthesis of these bioactive compounds and endogenous phytohormones, as well as their associated pathways. This study provides a theoretical and practical basis for understanding the physiological and transcriptional responses underlying polyphenols and triterpenoids biosynthesis in *P. vulgaris* following exogenous SNP treatment. Furthermore, this study provides a foundation for future field studies on the understory ecological cultivation of *P. vulgaris* and suggests a promising strategy for exogenous SNP application to improve the content of bioactive compounds in medicinal material.

## 2. Results

### 2.1. Physiological and Active Component Analysis

#### 2.1.1. NO and H_2_O_2_ Contents and Antioxidant Systems

**Figure 1:**
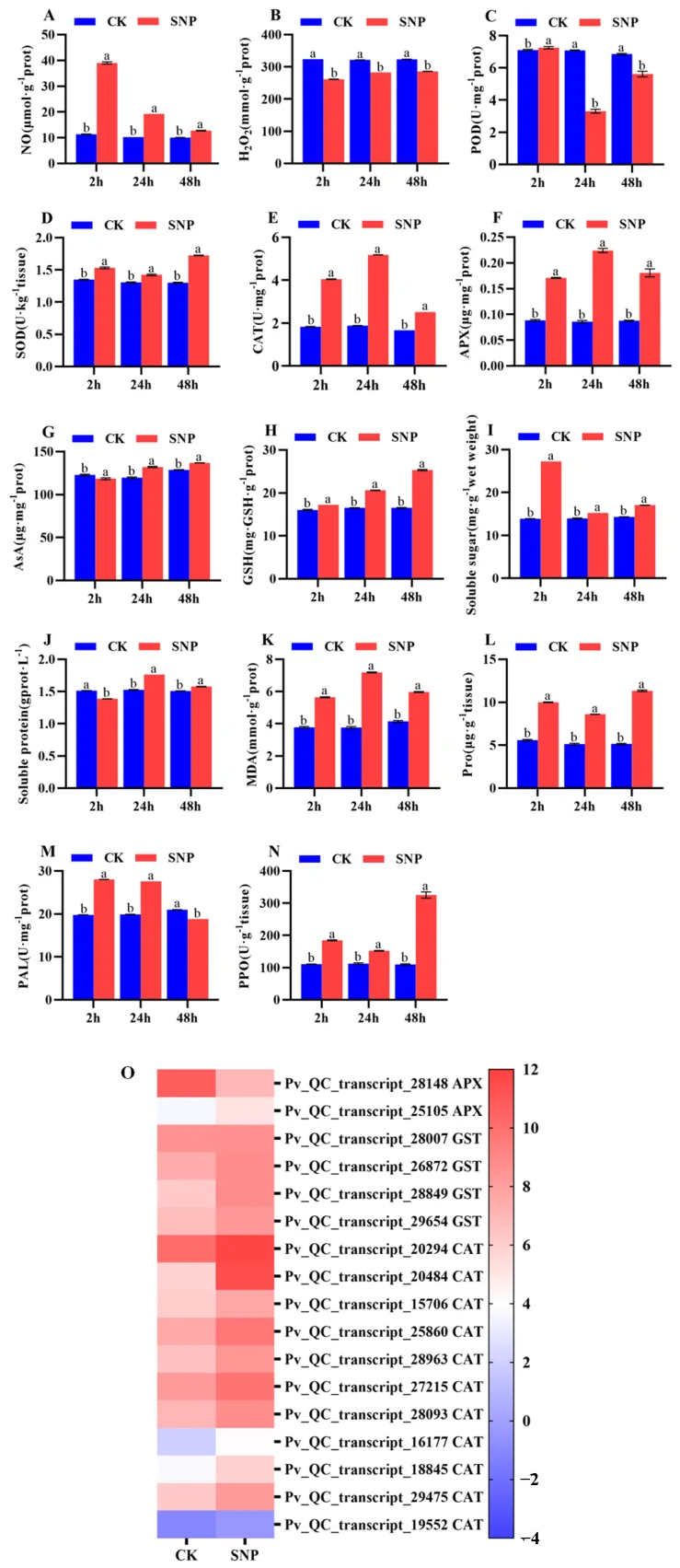
Influence of 100 μM exogenous SNP treatment on physiological and biochemical parameters in *P. vulgaris* seedling leaves at 2, 24, and 48 h. (**A**) Nitric oxide (NO) level. (**B**) Hydrogen peroxide (H_2_O_2_) level. (**C**) Peroxidase (POD) activity. (**D**) Superoxide dismutase (SOD) activity. (**E**) Catalase (CAT) activity. (**F**) Ascorbate peroxidase (APX) activity. (**G**) Ascorbic acid (AsA) level. (**H**) Glutathione (GSH) level. (**I**) Soluble sugar level. (**J**) Soluble protein level. (**K**) Malondialdehyde (MDA) level. (**L**) Proline (Pro) level. (**M**) Phenylalanine ammonia-lyase (PAL) activity. (**N**) Polyphenol oxidase (PPO) activity. (**O**) Heatmap of log_2_ (FPKM + 1)-transformed values for DETs encoding APX, CAT, and glutathione S-transferase (GST) in control (CK) versus SNP-treated leaves at 24 h. The values are presented as the mean ± SD (*n* = 3). For panels (**A**–**N**), different lowercase letters above the bars indicate significant differences among treatments at each time point (*p* < 0.05), as determined by one-way ANOVA with LSD test. FPKM values were used only for heatmap visualization.

NO and H_2_O_2_ levels were determined in *P. vulgaris* seedling leaves subjected to 100 μM SNP or distilled water (control) at 2, 24, and 48 h post treatment (Figure 1A,B). Compared with the control treatment, exogenous SNP treatment significantly increased the leaf NO content by approximately 243.6%, 88.0%, and 26.8% at 2, 24, and 48 h, respectively (Figure 1A). Conversely, compared with that in the control plants, the H_2_O_2_ content in the leaves in the SNP-treated plants was significantly reduced by approximately 19.2%, 12.0%, and 11.6% at the corresponding time points (Figure 1B).

POD, SOD, CAT, and APX activities were measured in *P. vulgaris* seedling leaves following SNP treatment (Figure 1C–F). Compared with that of the control, POD activity increased modestly by 2.0% at 2 h, followed by significant decreases of 53.4% at 24 h and 18.2% at 48 h after SNP application (Figure 1C). SOD activity significantly increased by 13.6%, 9.0%, and 32.5% at 2, 24, and 48 h, respectively (Figure 1D). CAT activity increased more prominently, increasing by 121.0%, 175.4%, and 50.7% at the same time points (Figure 1E). Similarly, the APX activity increased substantially by 93.3%, 161.3%, and 105.3% at 2, 24, and 48 h post treatment, respectively (Figure 1F).

The AsA content in *P. vulgaris* seedling leaves decreased slightly, by approximately 3.7%, at 2 h after SNP treatment. In contrast, the AsA content increased significantly by 10.4% and 6.4% at 24 h and 48 h, respectively, compared with that in the control (Figure 1G). Furthermore, the GSH content was significantly increased by 7.3%, 24.7%, and 53.0% at 2 h, 24 h, and 48 h, respectively, in the SNP-treated plants compared with that in the untreated control plants (Figure 1H).

Gene Ontology (GO) enrichment analysis of the DETs revealed a significant overrepresentation of terms related to “binding” and “catalytic activity” in the molecular function category (Supplementary Figure S2). These broad GO terms may reflect a general transcriptional response to SNP treatment, although they are not specifically indicative of antioxidant capacity. Consistent with this functional enrichment and the observed increase in enzymatic activity, the expression of key antioxidant system-related transcripts was markedly upregulated at 24 h. Specifically, the transcript levels of 11 transcripts encoding catalase (*PvCAT*, EC:1.11.1.6), 4 transcripts encoding glutathione S-transferase (*PvGST*, EC:2.5.1.18), and 2 transcripts encoding ascorbate peroxidase (*PvAPX*, EC:1.11.1.11) were significantly greater in the SNP-treated leaves than in the control (Figure 1O). This coordinated upregulation at the transcriptional level provides direct molecular support for the increased activities of CAT and APX, as well as for the elevated GSH pool associated with GST-mediated metabolism.

#### 2.1.2. Soluble Sugar, Soluble Protein, MDA, and Pro Contents

To evaluate how *P. vulgaris* seedlings adjust their osmotic balance under SNP treatment, we quantified the levels of soluble sugars, soluble protein, MDA, and Pro (Figure 1I–L). Compared with the control treatment, exogenous SNP treatment significantly increased the leaf soluble sugar content by 95.9%, 9.2%, and 19.2% at 2, 24, and 48 h, respectively (Figure 1I). The soluble protein content showed a biphasic response across the sampling time points: it initially decreased by 8.4% at 2 h but subsequently increased significantly by 15.5% and 4.6% at 24 and 48 h, respectively (Figure 1J). The MDA content, a marker of lipid peroxidation, and the Pro content, an osmotic adjustment substance, both substantially increased. The MDA content increased by 49.8%, 91.0%, and 44.3% at 2, 24, and 48 h, respectively, while the Pro content increased by 79.0%, 67.9%, and 120.3%, respectively, at the same time points (Figure 1K,L).

#### 2.1.3. PAL and PPO Activities

The effects of exogenous SNP on PAL and PPO activities in *P. vulgaris* seedling leaves are shown in Figure 1M,N. PAL activity showed distinct changes at the three sampling time points. It was significantly increased by 41.7% and 38.8% at 2 and 24 h post treatment, respectively, in the SNP-treated groups. However, by 48 h, the activity was significantly reduced by 10.3% compared with that of the control (Figure 1M). In contrast, PPO activity showed a sustained and marked increase at all the observed time points. It was elevated by 66.9%, 35.5%, and 196.2% at 2, 24, and 48 h, respectively, in the SNP-treated plants compared with the untreated controls (Figure 1N).

#### 2.1.4. Phytohormone Levels and Metabolism-Associated Transcripts

**Figure 2:**
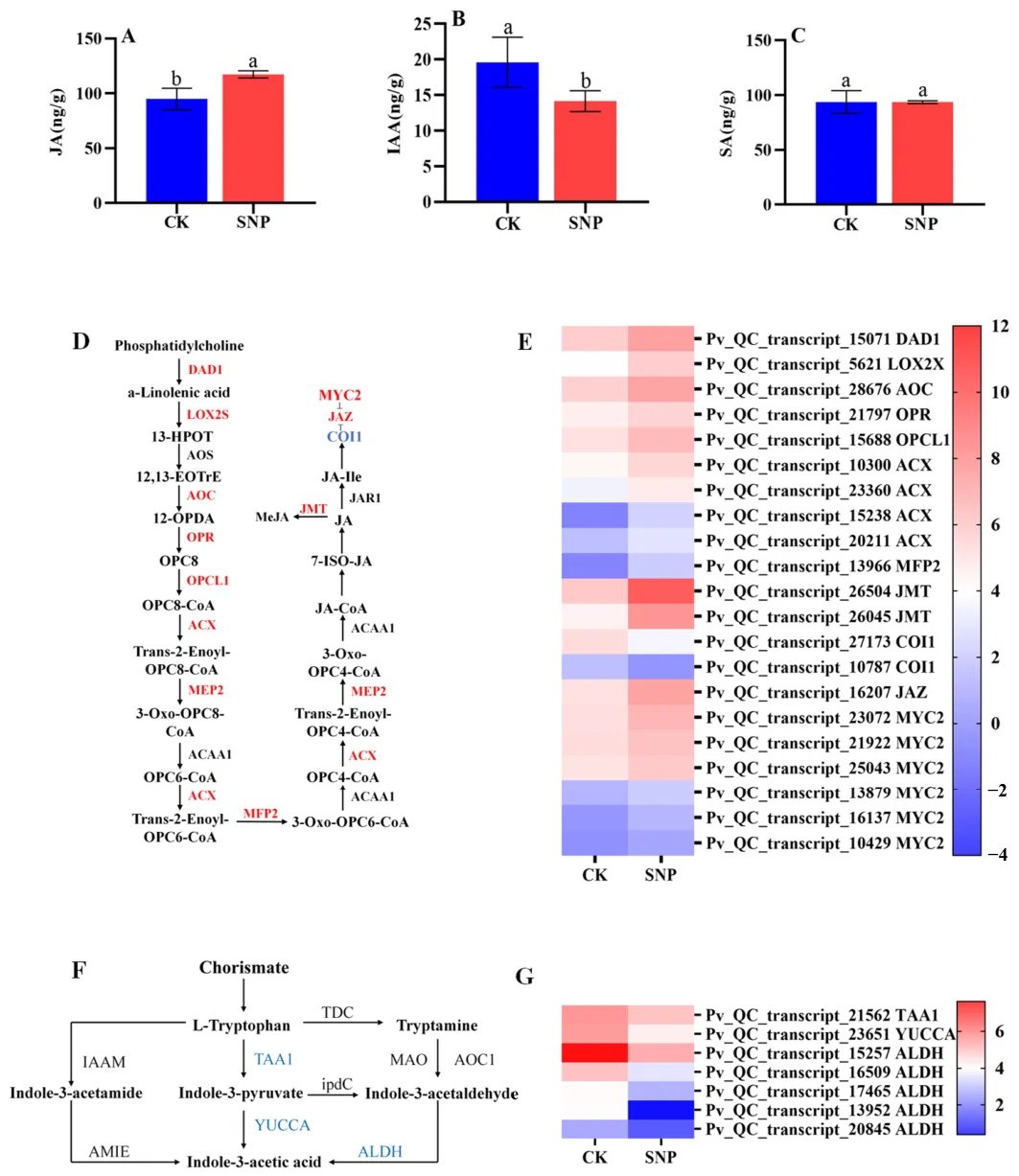
Influence of 100 μM exogenous SNP on endogenous phytohormone levels and related transcriptional changes in *P. vulgaris* seedling leaves 24 h after treatment. (**A**) Jasmonic acid (JA) level. (**B**) Indole-3-acetic acid (IAA) level. (**C**) Salicylic acid (SA) level. The values are expressed as the mean ± SD (*n* = 3). Significant differences between treatments at *p* < 0.05 are denoted by different lowercase letters above the bars (one-way ANOVA with LSD test). (**D**) Schematic of the JA biosynthesis and signaling pathway. (**E**) Heatmap indicating log_2_ (FPKM + 1)-transformed values for DETs related to JA metabolism and signaling. (**F**) Schematic of the IAA biosynthesis pathway. (**G**) Heatmap of log_2_ (FPKM + 1)-transformed values for DETs involved in IAA biosynthesis. In (**E**,**G**), red and blue boxes indicate upregulated and downregulated DETs, respectively. FPKM values were used only for heatmap visualization. JA pathway: DAD1, DEFECTIVE IN ANTHER DEHISCENCE 1 (phospholipase A1); LOX2S, lipoxygenase 2S; AOC, allene oxide cyclase; OPR, 12-oxophytodienoate reductase; OPCL1, OPC-8:0 CoA ligase 1; ACX, acyl-CoA oxidase; MFP2, multifunctional protein 2 (in β-oxidation); JMT, jasmonic acid carboxyl methyltransferase; MYC2, transcription factor MYC2; JAZ, jasmonate ZIM-domain protein; COI1, coronatine insensitive 1. IAA pathway: TAA1, tryptophan aminotransferase of Arabidopsis 1; YUCCA, YUCCA flavin-containing monooxygenase; ALDH, aldehyde dehydrogenase.

Endogenous JA, SA, and IAA levels in *P. vulgaris* leaves were quantified 24 h after treatment with 100 μM SNP (Figure 2A–C). Compared with the control group, the JA content was significantly increased by 23.5%, whereas the IAA content was significantly decreased by 27.8% (Figure 2A,B). However, the SA content did not differ significantly between the two groups (Figure 2C).

The elevated JA level was accompanied by the coordinated upregulation of transcripts encoding key biosynthetic enzymes, including lipoxygenases (LOXs), allene oxide cyclase (AOC), and acyl-CoA oxidase (ACX) (Figure 2E). Notably, transcripts involved in JA signaling and metabolism exhibited a complex expression pattern: while the transcription factor MYC2 was upregulated, we also observed the upregulation of jasmonate ZIM-domain proteins (JAZs, typical transcriptional repressors) and the downregulation of the receptor CORONATINE INSENSITIVE 1 (COI1) (Figure 2D). This pattern may suggest a potential feedback regulation or adaptation mechanism in the JA signaling pathway following SNP treatment.

The reduction in IAA content was correlated with the significant downregulation of transcripts encoding key biosynthetic enzymes, notably TAA1 and several YUCCA and ALDH family transcripts (Figure 2F,G). Consistent with the stable physiological level of SA, no significant differential expression was observed in transcripts involved in the core SA biosynthesis and signaling pathways at this time point.

#### 2.1.5. Secondary Metabolite Contents and Transcriptional Regulation of Their Biosynthesis

**Figure 3:**
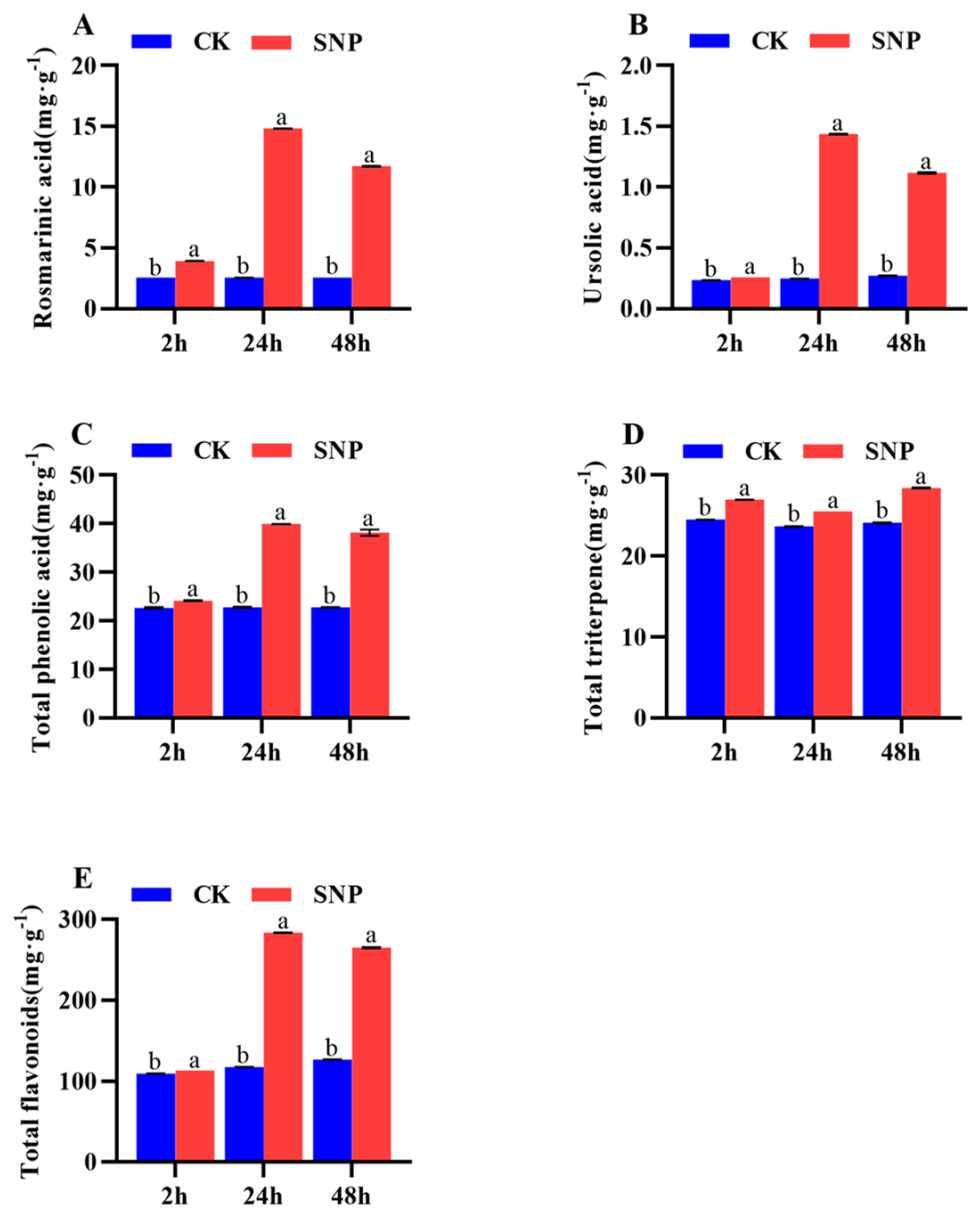
Influence of 100 μM exogenous SNP on specific and total bioactive compound levels in *P. vulgaris* seedling leaves at 2, 24, and 48 h. (**A**) Rosmarinic acid (RA) level. (**B**) Ursolic acid (UA) level. (**C**) Total phenolic acid level. (**D**) Total triterpenoid level. (**E**) Total flavonoid level. The data are expressed as the mean ± SD (*n* = 3). For panels (**A**–**E**), different lowercase letters above the bars indicate significant differences among treatments at each time point (*p* < 0.05), as determined by one-way ANOVA with LSD test.

Treatment with 100 μM SNP significantly promoted the accumulation of key bioactive compounds in *P. vulgaris* leaves over a 48-h period. The content of RA increased substantially, by approximately 53.6%, 488.0%, and 361.5% at 2, 24, and 48 h after treatment, respectively, compared with that in the control (Figure 3A). Similarly, UA contents markedly increased, by approximately 10.0%, 488.4%, and 314.3% at the corresponding time points (Figure 3B). The total phenolic acid content was significantly increased by 6.6%, 75.4% and 67.7% at 2, 24 and 48 h, respectively (Figure 3C). The total triterpenoid content significantly increased by 10.0%, 7.9%, and 17.9% at 2, 24 and 48 h, respectively (Figure 3D). Furthermore, the total flavonoid content substantially increased by 3.6%, 141.7% and 109.5% at 2, 24 and 48 h, respectively (Figure 3E).

**Figure 4:**
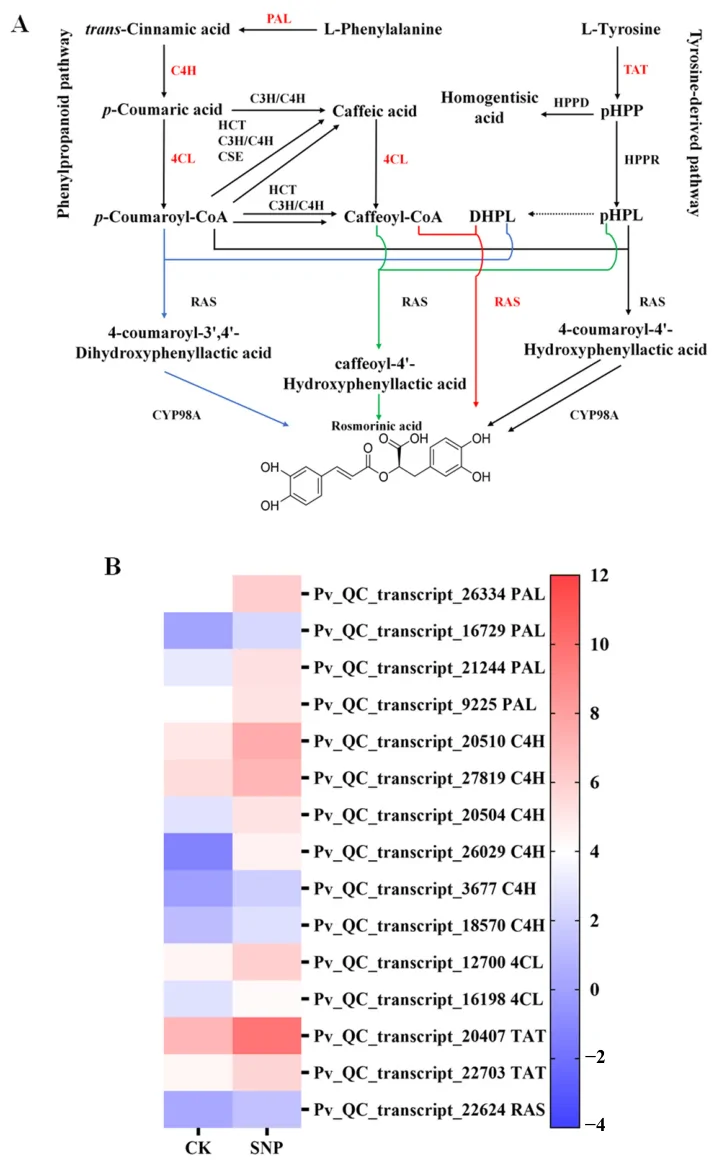

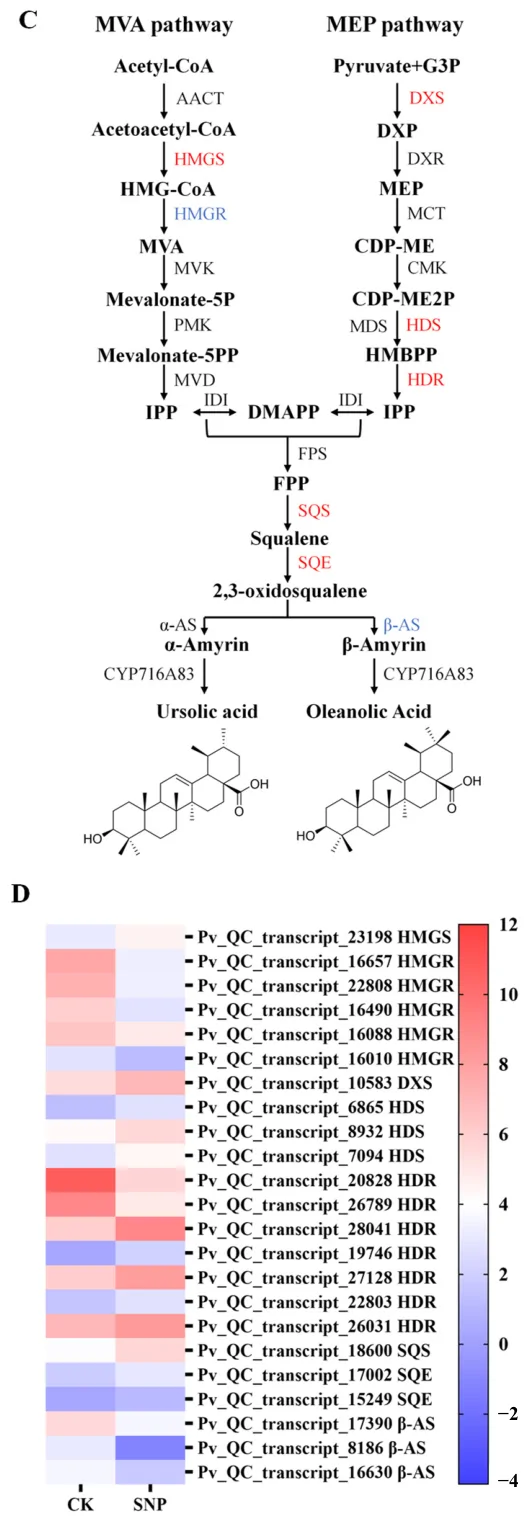
Transcriptional changes in key secondary metabolic pathways in *P. vulgaris* seedling leaves in response to 100 μM SNP treatment for 24 h. (**A**) Schematic of the phenylpropanoid/RA biosynthesis pathway, with DETs highlighted. The upregulated and downregulated transcripts are marked by red and blue boxes, respectively. (**B**) Expression heatmap showing log_2_ (FPKM + 1)-transformed values of DETs related to polyphenolic biosynthesis. (**C**) Schematic overview of the terpenoid backbone/UA biosynthesis pathway with the DETs highlighted. (**D**) Expression heatmap indicating log_2_ (FPKM + 1)-transformed values of DETs associated with triterpenoid biosynthesis. FPKM values were used only for heatmap visualization.

Transcriptomic analysis was conducted to explore the transcriptional changes associated with the observed metabolic enhancement. KEGG pathway enrichment analysis revealed significant enrichment of the phenylalanine/tyrosine metabolism pathway, which encompasses RA biosynthesis (Figure 4A). In agreement with RA accumulation, multiple transcripts encoding key biosynthetic enzymes in this pathway were coordinately upregulated (Figure 4B), including four *PvPAL* transcripts, six *PvC4H* transcripts, two *Pv4CL* transcripts, two *PvTAT* transcripts, and one *PvRAS* transcript.

Similarly, the accumulation of UA was associated with transcriptional changes in terpenoid biosynthesis (Figure 4C). The methylerythritol phosphate (MEP) pathway showed upregulation of one *PvDXS*, three *PvHDS*, and seven *PvHDR* transcripts. In contrast, the mevalonate (MVA) pathway exhibited downregulation of five *PvHMGR* transcripts, while one *PvHMGS* transcript was upregulated. We hypothesize that this metabolic shift may potentially favor the supply of precursors for UA synthesis. Furthermore, the upregulation of downstream transcripts (one *PvSQS* and two *PvSQE*) was consistent with increased UA biosynthesis, whereas the downregulation of three *Pvβ-AS* transcripts may potentially redirect metabolic flux toward UA rather than toward oleanolic acid (OA) (Figure 4D).

#### 2.1.6. In Vitro Antioxidant Capacity

**Figure 5:**
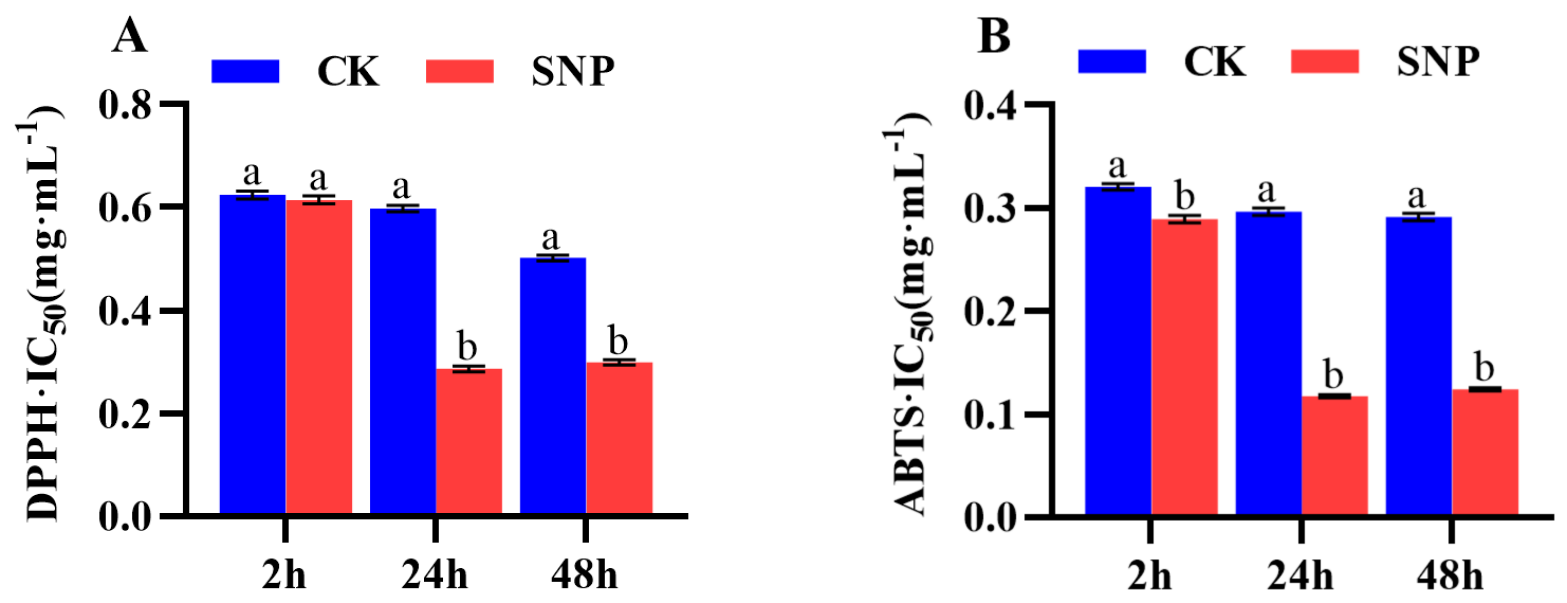
Influence of exogenous 100 μM SNP treatment on the in vitro antioxidant capacity of *P. vulgaris* leaf ethanol extracts at 2, 24, and 48 h post treatment. (**A**) DPPH radical scavenging activity, expressed as the half-maximal inhibitory concentration (IC_50_). (**B**) ABTS radical scavenging activity, expressed as the IC_50_. For panels (**A**,**B**), different lowercase letters above the bars indicate significant differences among treatments at each time point (*p* < 0.05), as determined by one-way ANOVA with LSD test.

The in vitro antioxidant capacity of *P. vulgaris* leaf ethanol extracts in response to 100 μM SNP treatment was evaluated over 48 h (Figure 5A,B). In the DPPH assay, there was no obvious difference between the control and SNP-treated samples at 2 h. At 24 h and 48 h, however, the IC_50_ values of the treated group decreased markedly to approximately 0.29 mg mL^−1^, i.e., 52.0% and 40.3% lower than those of the control group, respectively, indicating a substantial increase in antioxidant activity (Figure 5A). Moreover, compared with that in the control group, the ABTS radical scavenging capacity in the SNP-treated group was notably greater at all time points (Figure 5B). Specifically, the IC_50_ values of the treated group were reduced by 9.8% (2 h), 60.5% (24 h), and 57.4% (48 h) relative to the corresponding control values.

### 2.2. Transcriptome Analysis

#### 2.2.1. Transcriptome Sequencing and Transcriptome Assembly of *P. vulgaris*

To explore how exogenous SNP treatment affects *P. vulgaris* leaves, full-length transcriptome sequencing was conducted on samples treated with 100 μM SNP and distilled water (control) for 24 h. PacBio Sequel platform sequencing generated 353,897 polymerase reads, amounting to 50.45 Gb of raw data, with an average polymerase read length of 142,563 bp and an N50 value of 210,376 bp (Supplementary Table S1). After CCS correction, 298,991 CCS reads were obtained, with an average length of 2239 bp and an N50 of 2533 bp; among these, 280,786 FLNC reads were identified, with an average length of 2151 bp and an N50 of 2459 bp (Supplementary Table S2).

To increase data accuracy, the full-length transcriptome was subjected to multiple rounds of correction and redundancy removal. The number and length distribution of the transcripts before and after correction are summarized in Supplementary Table S3. After correction, 30,160 high-confidence nonredundant transcripts were obtained, with an average length of 2212 bp and an N50 of 2478 bp, and most transcripts fell within the 1–3 kbp range. These nonredundant transcripts were used as the reference for quantification, and they are referred to as transcripts rather than genes throughout this study.

#### 2.2.2. Analysis of DETs

**Figure 6:**
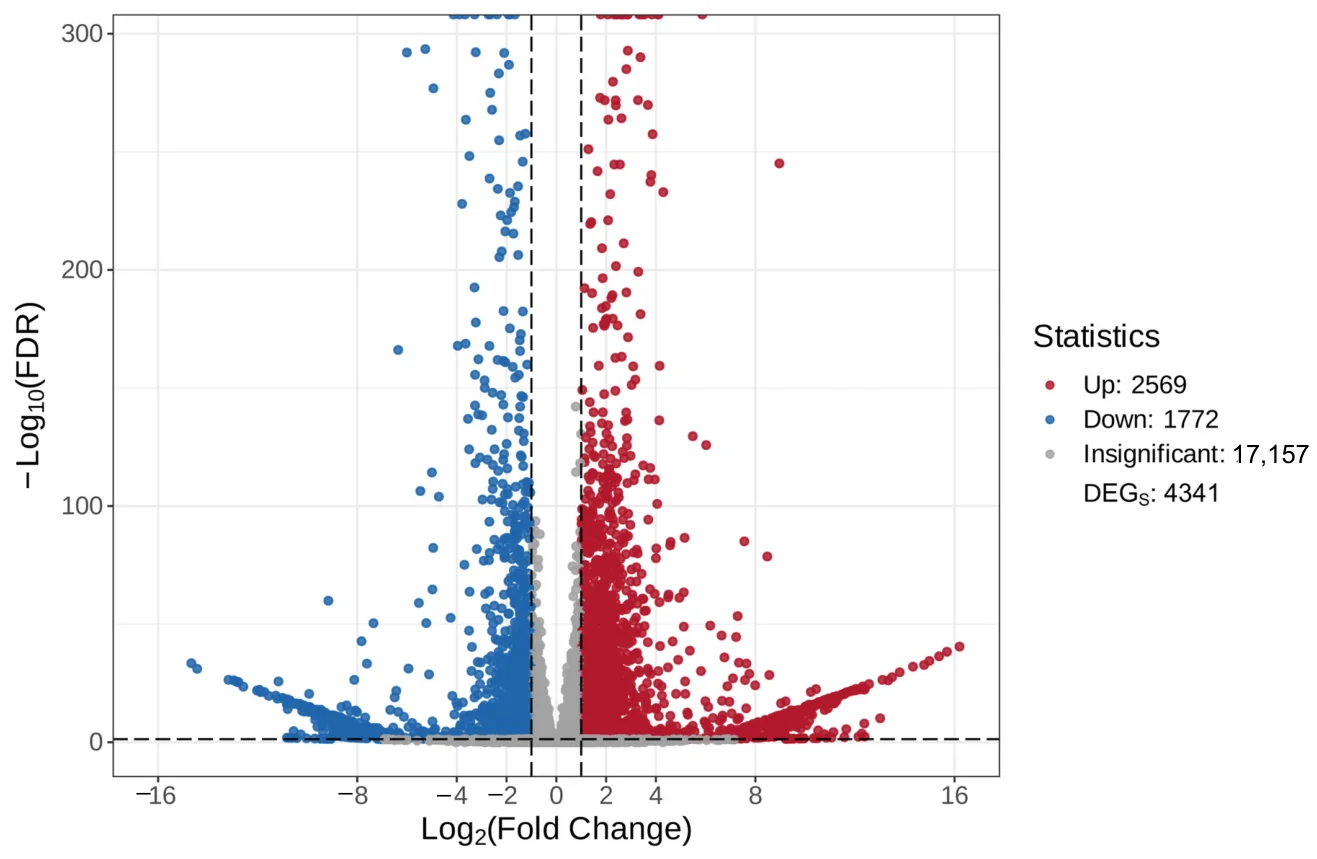
Volcano plot of DETs in *P. vulgaris* seedling leaves between the control (CK) and 100 μM SNP-treated groups at 24 h.

The transcriptome profiles of *P. vulgaris* seedling leaves from the control (CK) and 100 μM SNP-treated groups, each with three biological replicates, were compared at 24 h post treatment. Using DESeq2 (v1.40.2) with thresholds of FDR < 0.01 and |log_2_(fold change)| ≥ 1, based on raw count data, 4341 DETs were identified in total. Among these transcripts, 2569 transcripts were significantly upregulated and 1772 transcripts were downregulated in the SNP-treated group compared with the CK group (Figure 6).

GO enrichment analysis was performed to characterize the functional roles of the DETs between the SNP-treated and control groups. A total of 3399, 3584, and 3521 DETs were categorized into the biological process (BP), cellular component (CC), and molecular function (MF) categories, respectively (Supplementary Figure S2). In the BP category, DETs were enriched mainly in fundamental processes, including cellular processes, metabolic processes, and responses to stimuli. The CC category was dominated by terms related to cellular anatomical entities, whereas the MF category was primarily associated with binding and catalytic activity, which constitute the core functional set. These findings collectively indicate that SNP treatment triggers extensive transcriptional changes in *P. vulgaris* seedling leaves, significantly affecting cellular architecture, metabolic pathways, and stimulus-response mechanisms.

**Figure 7:**
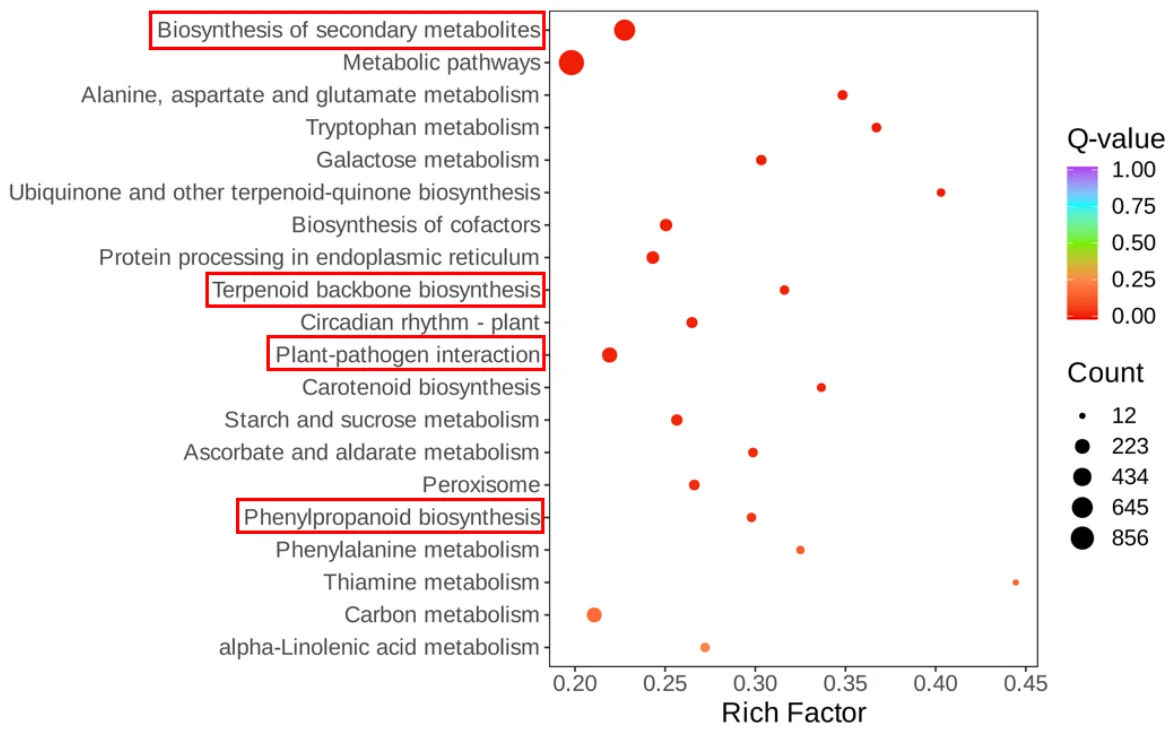
The top 20 KEGG pathways enriched among the upregulated DETs between the control (CK) and 100 μM SNP-treated groups in *P. vulgaris* seedling leaves at 24 h post treatment. The red box highlights, from top to bottom, secondary metabolite biosynthesis, terpenoid backbone biosynthesis, plant-pathogen interactions, and phenylpropanoid biosynthesis.

To investigate metabolic pathways and transcript functions, we found that 1222 upregulated DETs were annotated to 140 KEGG pathways between the CK and SNP groups at 24 h. Figure 7 presents the top 20 enriched pathways; among these, “secondary metabolite biosynthesis”, “phenylpropanoid biosynthesis”, “terpenoid backbone biosynthesis”, and “plant-pathogen interaction”, which are closely associated with secondary metabolism and defense-related pathways, contained the most upregulated DETs. Additionally, 65, 45, 47, and 129 DETs were significantly upregulated in the polyphenol-related, terpenoid-related, plant hormone signal transduction, and plant–pathogen interaction pathways, respectively (Supplementary Table S4).

#### 2.2.3. Expression Profiles of Transcription Factors (TFs)

**Figure 8:**
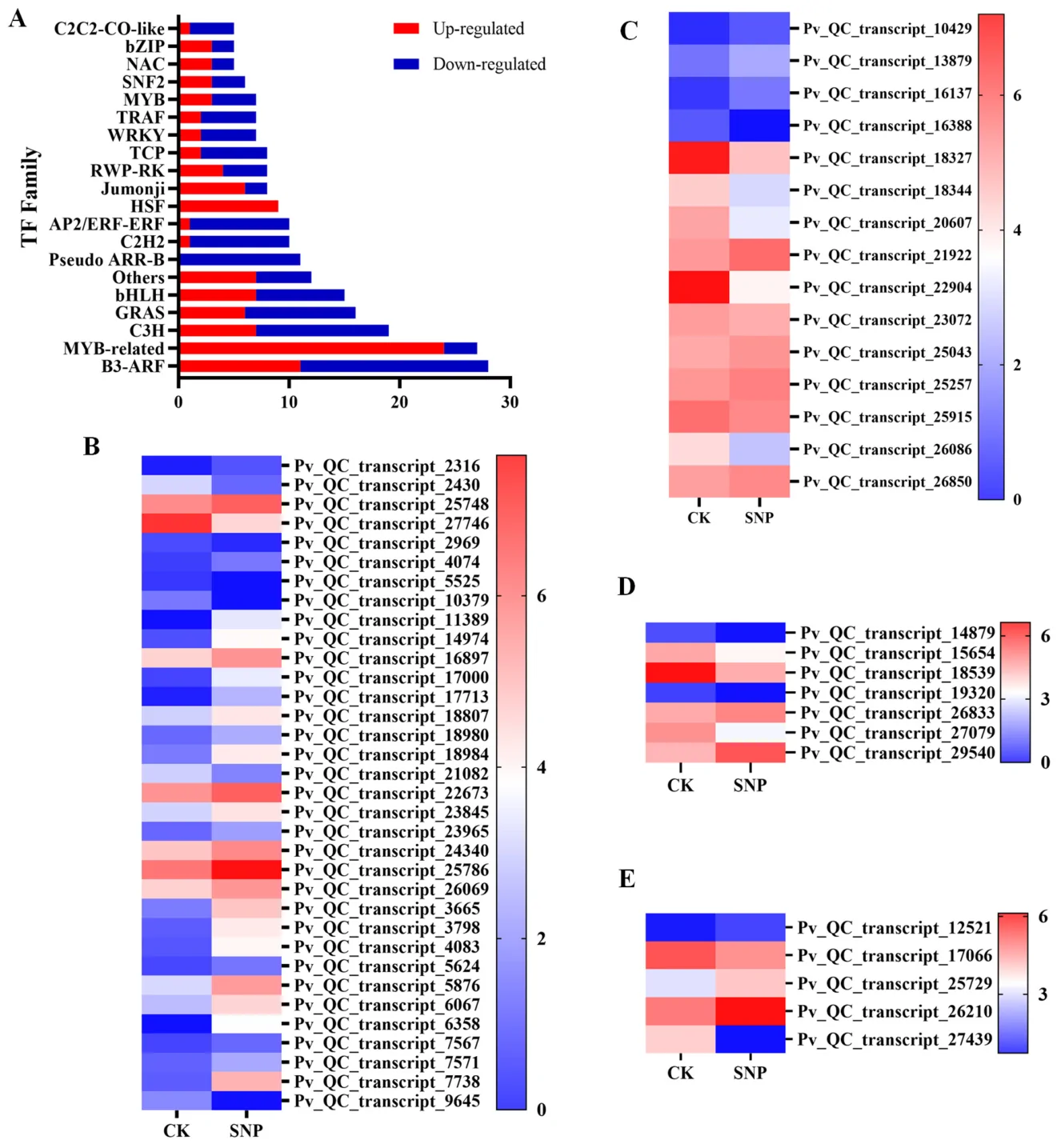
Differential expression of transcription factors (TFs) in response to exogenous SNP treatment in *P. vulgaris* seedling leaves. (**A**) Number of differentially expressed TFs between the CK and 100 μM SNP-treated groups at 24 h (FDR < 0.01, |log_2_FC| ≥ 1). (**B**–**E**) Expression heatmaps of key TF families (MYB/MYB-related, bHLH, WRKY, and NAC) based on log_2_(FPKM + 1) values. Red and blue colors indicate higher and lower expression levels, respectively. FPKM values were used only for heatmap visualization.

TFs play important roles in transcriptional responses to environmental stress. Comparative analysis revealed 295 differentially expressed TFs belonging to 54 families between the control and SNP-treated groups (136 upregulated and 159 downregulated). The top 20 are presented in Figure 8A. The most prominently altered families included MYB-related (24 upregulated, 3 downregulated), MYB (3 upregulated, 4 downregulated), bHLH (7 upregulated, 8 downregulated), WRKY (2 upregulated, 5 downregulated), and NAC (3 upregulated, 2 downregulated) (Figure 8B–E). Together, these results suggest that SNP treatment is associated with changes in the expression of key TF families, which may contribute to the transcriptional changes in secondary metabolism.

#### 2.2.4. RT-qPCR Validation of the RNA-Seq Data

**Figure 9:**
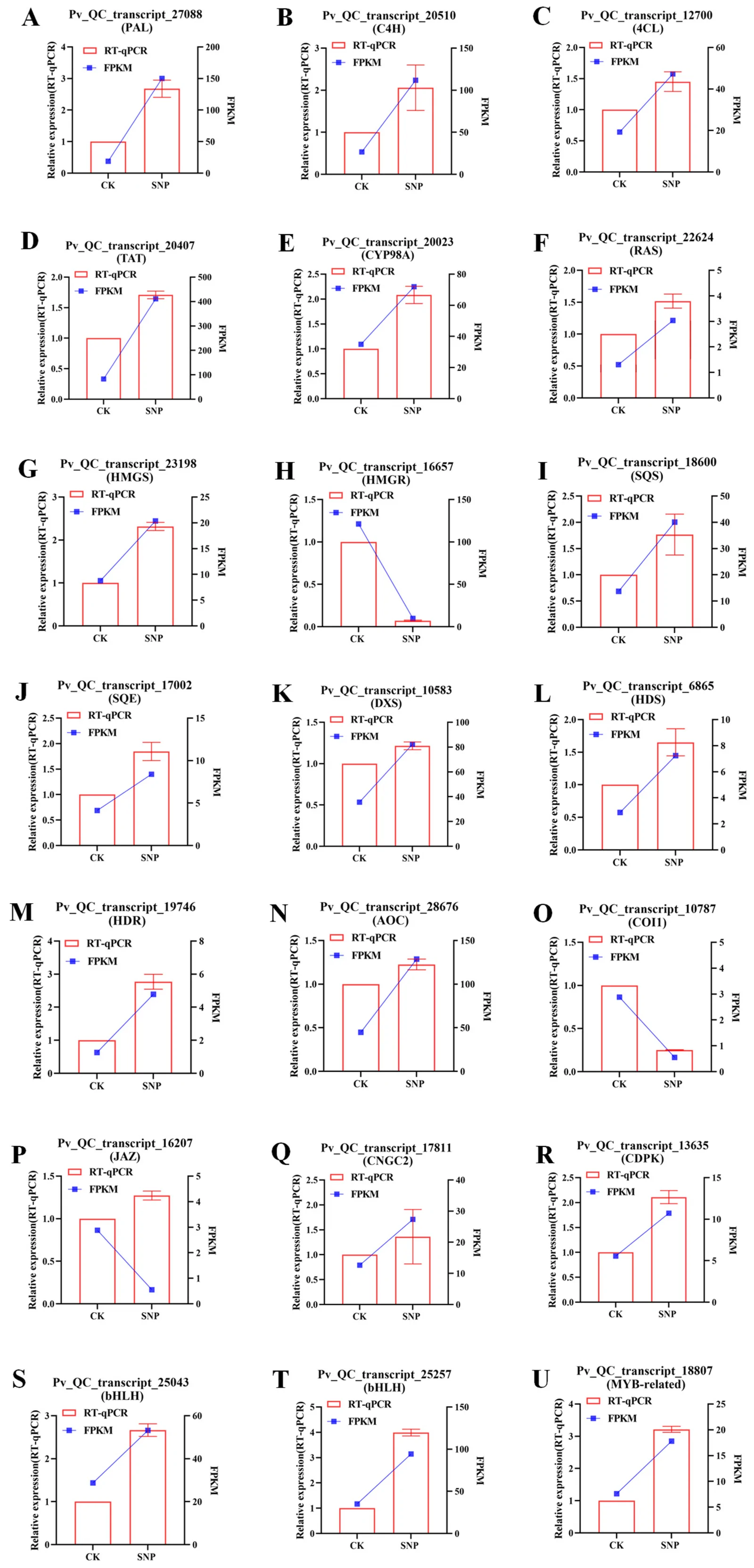
RT-qPCR validation of the RNA-Seq data. Relative expression (RT-qPCR) and log_2_ (FPKM + 1) values (RNA-Seq) of key transcripts related to polyphenol, terpenoid, and hormone signaling in the leaves of CK- and SNP-treated *P. vulgaris* seedlings after 24 h. (**A**) *PAL*, (**B**) *C4H*, (**C**) *4CL*, (**D**) *TAT*, (**E**) *CYP98A*, (**F**) *RAS*, (**G**) *HMGS*, (**H**) *HMGR*, (**I**) *SQS*, (**J**) *SQE*, (**K**) *DXS*, (**L**) *HDS*, (**M**) *HDR*, (**N**) *AOC*, (**O**) *COI1*, (**P**) *JAZ*, (**Q**) *CNGC2*, (**R**) *CDPK*, (**S**) *bHLH*, (**T**) *bHLH*, (**U**) *MYB-related*. Data are presented as the mean ± SD (*n* = 3).

To verify the reliability of the transcriptome results, the expression patterns of 21 selected DETs were examined using RT-qPCR. The qPCR profiles closely matched the RNA-Seq trends for all tested transcripts (Figure 9), including six transcripts encoding key enzymes involved in RA biosynthesis (*PAL*, *C4H*, *4CL*, *TAT*, *CYP98A*, and *RAS*); seven terpenoid biosynthesis-related transcripts (*HMGS*, *HMGR*, *SQS*, *SQE*, *DXS*, *HDS*, and *HDR*); three transcripts associated with the JA signaling pathway (*AOC*, *COI1*, and *JAZ*); two defense signaling pathway transcripts (*CNGC2* and *CDPK*); and three transcription factor transcripts (two *bHLH* and one *MYB-related*). Collectively, these findings support the reliability of the sequencing data and confirm the transcriptional changes associated with SNP treatment.

## 3. Discussion

### 3.1. NO Levels, H_2_O_2_ Contents, and Antioxidant Systems

Exogenous SNP treatment is associated with changes in antioxidant signaling in *P. vulgaris*, thereby affecting the plant’s defense against oxidative stress. As a well-known NO donor, SNP can release NO to participate in plant physiological regulation. NO is a crucial signaling molecule that regulates plant growth and development and responds to diverse biotic and abiotic stresses [33,34]. After foliar spraying with 100 μM exogenous SNP, the endogenous NO content in *P. vulgaris* seedling leaves significantly increased at 2, 24, and 48 h post treatment (Figure 1A), which aligns with findings from previous studies [27,35].

Studies have shown that exogenous NO can modulate the activities of enzymes associated with NO synthesis, such as nitric oxide synthase (NOS) in plants (e.g., wheat seedling leaves), affect the transcription levels of key nitrogen assimilation transcripts (such as NR, NiR, and GS), promote the synthesis of endogenous NO, or inhibit NO catabolism [36,37]. Notably, exogenous NO may inhibit the production of endogenous H_2_O_2_, induce plants to increase antioxidant defense system activity, upregulate the activity of antioxidant enzymes, and promote H_2_O_2_ decomposition [38] (Figure 1B). Specifically, foliar spraying with 100 μM SNP significantly increased the activities of SOD, CAT, and APX in *P. vulgaris* seedling leaves at 2, 24, and 48 h (Figure 1D–F). This increase may be linked to the significant upregulation of antioxidant enzyme-encoding transcripts, as revealed by our transcriptome data. After 24 h of SNP treatment, 11 CAT transcripts and 2 APX transcripts were significantly upregulated in *P. vulgaris* seedling leaves, and the increased activity of these antioxidant enzymes may have contributed to the lower H_2_O_2_ levels observed at 2, 24, and 48 h post SNP treatment (Figure 1B,O).

Notably, an interesting phenomenon was observed: POD activity in the SNP group increased at 2 h but was significantly lower than that in the control (CK) group at 24 and 48 h (Figure 1C), which may be related to the dynamic changes in the expression of POD-encoding transcripts in *P. vulgaris* seedling leaves under SNP treatment. Consistent with these findings, previous studies on medicinal plants have shown that POD transcript expression can be either upregulated or downregulated under oxidative stress; for example, POD transcript expression is significantly upregulated in *S. miltiorrhiza* under drought-induced oxidative stress when *AtDREB1A* is overexpressed [39], whereas POD transcript expression is downregulated in *A. lancea* under prolonged drought stress [40].

In this study, GSH levels were significantly higher in *P. vulgaris* seedling leaves under SNP treatment than in the control from 2 to 48 h, which is consistent with the central role of GSH in the plant antioxidant defense system. Interestingly, the expression of 4 GST transcripts was significantly upregulated in SNP-treated leaves (Figure 1H,O); these enzymes can catalyze the combination of GSH with peroxide products, thereby increasing plant tolerance to stress [41]. Moreover, we found that although the AsA content decreased significantly at 2 h post treatment, it increased significantly at 24 and 48 h (Figure 1G). Research has indicated that SNP treatment can markedly increase AsA content, which may lead to reduced accumulation of H_2_O_2_ and MDA under SNP treatment [37]. Therefore, these results imply that SNP treatment, by affecting endogenous NO accumulation and H_2_O_2_ decomposition, significantly affects the NO and H_2_O_2_ contents and the enzymatic antioxidant system of *P. vulgaris* seedling leaves.

### 3.2. Osmotic Adjustment Substances

When plants are exposed to exogenous SNP treatment, soluble sugars, soluble protein, MDA, and Pro play important roles in maintaining cellular homeostasis and alleviating stress-induced damage [38]. Compared with those in the untreated control leaves, the MDA and Pro contents in *P. vulgaris* seedling leaves were notably greater at 2, 24, and 48 h post treatment, which is in line with our earlier study on *P. vulgaris* under oxidative stress [16] (Figure 1K,L). The elevated MDA level indicated lipid peroxidation damage in seedling leaves under SNP treatment, whereas the simultaneous accumulation of Pro reflected the active osmotic adjustment response of the plants. This phenomenon might be related to the enhanced activity of the antioxidant system in *P. vulgaris* seedlings after 2–48 h of SNP treatment, which can effectively scavenge reactive oxygen species (e.g., H_2_O_2_) induced by SNP treatment and mitigate oxidative damage to cellular membranes.

The soluble protein content in *P. vulgaris* seedling leaves decreased notably at 2 h after SNP application but increased markedly at 24 and 48 h (Figure 1J). There are two possible explanations for this dynamic change: on the one hand, at the early stage of SNP treatment (2 h), more free amino acids might be diverted to phenylpropanoid metabolism to synthesize secondary metabolites, thus reducing soluble protein accumulation and enabling the plant to rapidly defend against SNP-induced oxidative responses [42,43]; on the other hand, at 24–48 h, exogenous SNP significantly increased the soluble protein content, which may be associated with the increased endogenous NO levels and a potential reduction in the conversion of soluble protein to free amino acids, thereby maintaining cellular osmotic potential in *P. vulgaris* leaves and contributing to antioxidant capacity [2]. Moreover, we found that the soluble sugar content significantly increased at all time points (2–48 h) under the SNP treatment (Figure 1I). This accumulation of soluble sugars may contribute to the plant’s response to SNP treatment through dual mechanisms: first, sugars and phenolic compounds can work together to generate reactive oxygen species (ROS) in plant cells and modulate defense responses [44]; second, sugars not only directly take part in the synthesis of biochemical compounds and energy production but also play a key role in maintaining membrane structural stability under these conditions [45]. Collectively, these results demonstrated that *P. vulgaris* seedlings accumulate soluble sugars, soluble protein, and Pro, which may contribute to osmotic adjustment, while the increased MDA level indicates lipid peroxidation in response to SNP treatment. These physiological changes may help the plant cope with SNP-induced redox perturbations.

### 3.3. Secondary Metabolism-Related Enzyme Activities

PAL, a key enzyme in the phenylpropanoid metabolic pathway, is critical for driving phenylalanine catabolism and phenolic compound biosynthesis in *P. vulgaris* [46]. Exogenous SNP application can increase PAL activity in plants, which further accelerates the biosynthesis of polyphenolic components, thereby strengthening the ability of plants to resist oxidative damage and adapt to various abiotic stresses [27,47,48]. Our study revealed that 100 μM exogenous SNP significantly increased PAL activity in *P. vulgaris* seedling leaves at 2 and 24 h post treatment (Figure 1M). This dynamic change may be associated with the significant upregulation of 4 PAL-encoding transcripts in *P. vulgaris* leaves after 24 h of SNP treatment, which aligns with the findings of previous studies on medicinal plants under SNP treatment [6,49] (Figure 4B). Interestingly, PAL activity decreased significantly at 48 h after SNP treatment, which may suggest a potential feedback regulation when polyphenol levels reach a certain threshold, thereby modulating PAL transcript expression and activity to maintain metabolic balance [50,51].

Similarly, PPO activity and transcript expression levels in plant leaf tissues are closely associated with a reduction in abiotic stress damage, as PPO is also a critical enzyme involved in plant phenolic metabolism and defense responses [46]. In our research, SNP treatment significantly increased PPO activity in *P. vulgaris* seedling leaves at all time points (2, 24 and 48 h) (Figure 1N). This sustained increase may be associated with changes in PPO-encoding transcript expression under SNP treatment. Consistent with the findings of previous studies, enhanced PPO activity is closely associated with phenolic metabolism, thereby contributing to the plant’s response to SNP treatment [25,49]. Overall, the dynamic changes in PAL activity and persistent increase in PPO activity are closely tied to the physiological responses of *P. vulgaris* seedlings to SNP treatment.

### 3.4. Phytohormones and Their Metabolism-Related Transcripts

This study confirmed that foliar application of 100 μM exogenous SNP increased NO accumulation in *P. vulgaris* seedling leaves, which was accompanied by elevated JA synthesis and upregulated transcription of JA-related synthase transcripts at 24 h (Figure 2A). Further verification revealed that endogenous NO accumulation in these seedlings was closely associated with the upregulation of JA biosynthesis-related synthase transcripts, such as *PvDAD1*, *PvLOX2S*, *PvAOC*, *PvOPR*, *PvOPCL1*, *PvACX*, and *PvMFP2*. Notably, the transcript level of COI1 (a coreceptor in JA signaling) was suppressed, while JAZ transcripts were upregulated, suggesting a potential feedback regulation of JA signaling following SNP treatment (Figure 2D,E). Additionally, SNP-induced JA accumulation was accompanied by increased polyphenolic biosynthesis, as well as the transcription levels of phenylpropanoid synthases (e.g., *PvPAL*, *PvC4H*, *Pv4CL*, *PvTAT*, and *PvRAS*) and terpenoid synthases (e.g., *PvDXS*, *PvHDS*, *PvHDR*, *PvHMGS*, *PvSQS*, and *PvSQE*) (Figure 4A–D). These findings are similar to those of our early study, which revealed that H_2_O_2_ induces JA accumulation that is accompanied by the expression of polyphenol and triterpenoid metabolism-related transcripts in *P. vulgaris* leaves [16]. Overall, these findings suggest that exogenous SNP treatment is associated with JA accumulation and the synthesis of polyphenolic components (e.g., RA, phenolic acids, and flavonoids) and triterpenoids (e.g., UA) in *P. vulgaris* seedling leaves; however, whether JA directly mediates these metabolic changes requires further investigation.

The results of the present study revealed that compared with the control treatment, the increase in endogenous NO following SNP treatment was accompanied by a significant reduction in IAA content in *P. vulgaris* seedling leaves (Figure 2B). Two potential mechanisms may explain this phenomenon: First, 24 h of exogenous SNP treatment significantly decreased the IAA content, which may be associated with the suppressed transcription of IAA synthesis-related transcripts (e.g., *PvTAA1*, *PvYUCCA*, and *PvALDH*), thereby reducing IAA biosynthesis, which is consistent with previous research [52]. Second, plant phenolics can either restrict IAA synthesis or promote its catabolism [53]. These observations suggest a potential link between phenolic accumulation and reduced IAA levels, but the causal relationship remains to be established.

Finally, exogenous SNP did not significantly affect the SA content in *P. vulgaris* seedling leaves in this study (Figure 2C). Transcriptomic data suggested that this result may be related to the unchanged expression of SA biosynthesis-related transcripts. Furthermore, exogenous SA application has been shown to significantly reduce the polyphenol content (e.g., total phenolic acids, total flavonoids, and RA) in *P. vulgaris* spikes [2]. Similar results have been reported previously [54,55], but the specific regulatory mechanisms remain to be further explored. Collectively, these results indicate that SNP treatment is associated with the production of polyphenols (e.g., total phenolic acid, total flavonoids, and RA) and triterpenoids (e.g., UA), which occurs concurrently with suppressed IAA biosynthesis in *P. vulgaris* seedling leaves; however, the causal hierarchy among these changes requires further validation.

### 3.5. Secondary Metabolite Contents and Expression of Metabolism-Related Transcripts

In this study, compared with that in the CK group, the RA content in *P. vulgaris* seedling leaves was significantly elevated at 2–48 h after exogenous SNP treatment (Figure 3A). Transcriptomic analysis revealed that exogenous SNP treatment for 24 h was associated with increased expression of transcripts involved in the phenylalanine and tyrosine pathways of the RA synthesis pathway in *P. vulgaris*, including 4 upregulated *PvPAL* transcripts, 6 upregulated *PvC4H* transcripts, 2 upregulated *Pv4CL* transcripts, 2 upregulated *PvTAT* transcripts, and 1 upregulated *PvRAS* transcript (Figure 4A,B). Previous studies have demonstrated that the application of 200 μM SNP (as an NO donor) can increase flavonoid and phenolic compound contents (especially those of RA) in *S. miltiorrhiza* and increase the resistance of *S. miltiorrhiza* leaves to oxidative stress induced by copper [29]. Our earlier study also systematically characterized RA biosynthesis-related transcript families in *P. vulgaris* and verified the functional roles of *PvRAS* transcripts (particularly *PvRAS3* and *PvRAS4*), further supporting that SNP-induced PvRAS upregulation is associated with RA accumulation [1].

Moreover, compared with that in the CK group, the UA content in *P. vulgaris* seedling leaves significantly increased at 2–48 h post exogenous SNP treatment (Figure 3B). In the two terpenoid synthesis pathways, exogenous SNP treatment was associated with changes in the expression of key enzyme-encoding transcripts of the mevalonate (MVA) and methylerythritol phosphate (MEP) pathways, including 1 upregulated *PvDXS* transcript, 3 upregulated *PvHDS* transcripts, 7 upregulated *PvHDR* transcripts, 1 upregulated *PvHMGS* transcript, 1 upregulated *PvSQS* transcript, and 2 upregulated *PvSQE* transcripts (Figure 4C,D). Du 2015 [56] investigated the effects of NO on tanshinone accumulation in water stress-induced *S. miltiorrhiza* hairy roots and observed that NO signaling is involved in the MVA and MEP pathways, significantly stimulating the accumulation of 4 tanshinones and other terpenoids at 100 μM SNP. Similarly, Gu 2017 [57] reported that NO can upregulate the expression of key triterpenoid biosynthetic enzyme-encoding transcripts (including *SQS*, *HMGS*, and *DXS*) in submerged fermenting *Ganoderma lucidum*, thereby promoting triterpenoid accumulation.

Compared with the CK treatment, exogenous SNP treatment significantly increased the total phenolic and total flavonoid contents in *P. vulgaris* seedling leaves at 2, 24, and 48 h post treatment (Figure 3C,E). This polyphenolic (phenolic acids and flavonoids) accumulation may be explained by two factors: First, SNP treatment was associated with increased transcription of key transcripts involved in phenolic acid and flavonoid biosynthesis in *P. vulgaris* seedling leaves, including 4 upregulated *PvPAL* transcripts, 6 upregulated *PvC4H* transcripts, and 2 upregulated *Pv4CL* transcripts, which is consistent with polyphenolic synthesis, which is consistent with our earlier study on *P. vulgaris* under oxidative stress [16]. Second, SNP treatment may be associated with the activation of specific signal transduction pathways (e.g., the Ca^2+^ signaling pathway), which further promotes the transcription and expression of phenolic acid and flavonoid synthesis-related transcripts, directing more metabolic substrates toward phenolic acid and flavonoid synthesis and ultimately increasing polyphenolic content [47]. In addition, exogenous SNP treatment significantly increased the total triterpenoid content in *P. vulgaris* seedling leaves at all time points (2–48 h) (Figure 3D). This increased triterpenoid content may be due to SNP treatment upregulating the expression of triterpenoid biosynthesis-related metabolic enzyme-encoding transcripts, such as *PvHMGR*, *PvDXS*, *PvHDS*, and *PvHD*R, thereby promoting total triterpenoid accumulation (Figure 4C,D). Similar findings have been reported previously: Liu 2019 [58] observed that NO regulates triterpene acid accumulation by cooperating with H_2_O_2_ in *Ganoderma lingzhi*, and Zeng 2015 [59] demonstrated that NO crosstalks with Ca^2+^ and ROS to promote triterpenoid biosynthesis in *Betula platyphylla*.

Collectively, these results indicate that exogenous SNP treatment significantly increases the contents of polyphenolic components (e.g., RA, phenolic acids, and flavonoids) and terpenoids (e.g., UA) in *P. vulgaris* seedling leaves, which is closely associated with the upregulation of their respective biosynthesis-related transcripts.

### 3.6. In Vitro Antioxidant Activity

Consistent with the accumulation of polyphenolic and triterpenoid components following SNP treatment, in this study, the ethanol extracts of *P. vulgaris* seedling leaves exhibited strong DPPH and ABTS free radical scavenging capacity at 2–48 h after exogenous SNP treatment (Figure 5A,B). This enhanced in vitro antioxidant activity is likely attributed to the SNP-induced accumulation of polyphenolic (phenolic acids and flavonoids) and triterpenoid components in *P. vulgaris* seedling leaves, as these bioactive components are well known to be the main contributors to plant antioxidant capacity.

Numerous previous studies have confirmed the close association between polyphenolic compounds, triterpenoid components and antioxidant activity in medicinal plants. For instance, a recent study [60] evaluated the edible value of *S. miltiorrhiza* seeds and observed that the total phenolic acids, total flavonoids, and terpenoids in the seeds and aerial parts exhibited excellent scavenging capacities against ABTS and DPPH free radicals. Similarly, another study [61] isolated new constituents from *S. miltiorrhiza* aerial parts, verified their antioxidant potential, and further confirmed through metabolomic and antioxidant analyses that the seeds of *S. miltiorrhiza* and *Salvia prattii* possess strong antioxidant activity, which is closely related to their rich polyphenolic and terpenoid contents [62]. These results indicate that exogenous SNP treatment is associated with the accumulation of polyphenolic and triterpenoid components in *P. vulgaris* seedling leaves, which may contribute to the enhanced in vitro antioxidant activity.

Consistent with our findings, a previous study confirmed a strong positive correlation between the levels of RA and other phenolic acids in *Salvia* species and DPPH/ABTS antioxidant activity, identifying RA as a core contributor to the antioxidant capacity of Lamiaceae medicinal plants [63]. Further studies verified this in ethanol extracts of Lamiaceae plants (rosemary and thyme), showing that the DPPH/ABTS scavenging IC_50_ values were closely negatively correlated with the concentrations of polyphenols, flavonoids and triterpenoids, which is the key determinant of antioxidant activity in these plants [64]. In addition, previous research has confirmed that exogenous methyl jasmonate (MeJA) can effectively induce phenolic acid and terpenoid accumulation in *S. miltiorrhiza* and *P. vulgaris* through the upregulation of key transcripts involved in bioactive compound biosynthesis [2,65,66,67]. Collectively, these results indicate that exogenous SNP treatment for 24 h is associated with increased contents of bioactive components (e.g., total phenolic acids, total flavonoids, total triterpenoids, RA, and UA), which may contribute to the enhanced in vitro antioxidant activity in *P. vulgaris* seedling leaves.

### 3.7. Analyses of DET Expression Profiles

To explore the transcriptional changes associated with SNP treatment in *P. vulgaris* seedling leaves, we used transcriptome sequencing to examine transcript expression patterns 24 h after exogenous SNP treatment. In total, 4341 DETs were identified between the CK and SNP groups, with 2569 upregulated DETs and 1772 downregulated DETs (Figure 6). KEGG pathway annotation revealed that the three most abundant pathways were “biosynthesis of secondary metabolites”, “plant hormone signal transduction”, and “plant–pathogen interaction” (Supplementary Table S4), which is consistent with the known involvement of SNP in secondary metabolism and defense responses.

Many DETs were assigned to the “biosynthesis of secondary metabolites” pathway, which is associated with SNP treatment (Figure 7). Previous studies have confirmed that an increase in endogenous NO levels can increase the expression of polyphenol and terpenoid synthase transcripts, thereby facilitating the production of polyphenolic and terpenoid compounds [56,57]. Our research also confirmed that exogenous SNP treatment was associated with upregulated expression of phenylalanine and terpenoid synthase transcripts (e.g., *PvPAL*, *PvC4H*, *PvDXS*, and *PvHDS*), which was accompanied by the accumulation of polyphenols and triterpenoids in *P. vulgaris* seedling leaves (Figure 3).

Moreover, the “plant-pathogen interaction” pathway, mainly involving the Ca^2+^ signaling pathway, was also significantly enriched among the DETs. Specifically, 11 DETs encoding cyclic nucleotide-gated ion channels (CNGCs) were identified (6 upregulated, 5 downregulated), and 12 DETs encoding calcium-dependent protein kinases (CDPKs), of which 8 were upregulated and 4 were downregulated. Although no significant differential expression was observed for calmodulin (CALM/CML)-encoding transcripts in our transcriptomic data, the observed changes in CNGCs and CDPKs may indicate a perturbation of Ca^2+^ signaling in response to SNP treatment. Previous studies have demonstrated that exogenous NO interacts with CALM and CDPK to coregulate the accumulation of phenolic compounds and defense responses [68]. On the one hand, exogenous SNP treatment may increase intracellular NO levels, which may be associated with upstream Ca^2+^ signaling. This may further be associated with downstream antioxidant enzymes (e.g., APX and SOD) and secondary metabolism, thereby potentially contributing to the plant’s response to SNP treatment [69,70,71]. On the other hand, it is plausible that Ca^2+^ signaling, via CDPKs and potentially involving calmodulin-mediated pathways, may be associated with the expression of transcription factors such as MYBs, WRKYs, and NACs, which may contribute to the expression of stress resistance transcripts [71,72] or induce the expression of key enzyme-encoding transcripts in polyphenol and terpenoid synthesis pathways, ultimately being associated with the accumulation of active components such as RA and UA [73].

In addition, our study revealed that exogenous SNP supplementation was associated with the biosynthesis of phytohormones (e.g., JA) and polyphenolic and triterpenoid compounds in *P. vulgaris* seedling leaves (Figure 2A and Figure 3). Our previous studies also revealed that MeJA or H_2_O_2_ treatment was associated with the expression of transcripts related to polyphenol and triterpenoid biosynthesis, resulting in a significant increase in active components in the leaves and spikes of *P. vulgaris* [2,16]. These results indicate that exogenous SNP is associated with changes in the expression of transcripts related to endogenous hormones, Ca^2+^ signaling, and polyphenol and terpenoid biosynthesis in *P. vulgaris* seedling leaves at 24 h after treatment, which may collectively contribute to the observed metabolic responses.

### 3.8. SNP-Responsive TFs

In this study, 295 differentially expressed TF-encoding transcripts were identified between the control (CK) and SNP-treated groups at 24 h; these transcripts belonged primarily to the MYB-related/MYB, bHLH, WRKY, and NAC families (Figure 8). Specifically, MYB TF transcripts, the most prominent group among the differentially expressed TFs, were upregulated along with key polyphenol and triterpenoid biosynthetic transcripts, including *PvPAL*, *PvC4H*, *Pv4CL*, *PvTAT*, *PvRAS*, and *PvDXS,* in *P. vulgaris* seedling leaves. This expression pattern is consistent with the findings of previous studies on *S. miltiorrhiza* (a model medicinal plant of Lamiaceae), in which MYB TFs (e.g., SmMYB1 and SmMYB9b) have been demonstrated to mediate secondary metabolite accumulation [74,75]. Moreover, the Smi-miR858a-SmMYB module was recently shown to regulate tanshinone and phenolic acid biosynthesis by targeting SmMYB factors (e.g., SmMYB6, SmMYB97, and SmMYB112), which activate both direct biosynthetic transcripts and MeJA biosynthesis transcripts [76]. Similarly, bHLH TFs in *S. miltiorrhiza*, such as SmbHLH51 and SmbHLH148, promote polyphenol and terpenoid biosynthesis by upregulating the expression of polyphenol metabolic transcripts (*SmPAL*, *SmTAT*, *SmHPPR*, *Sm4CL*, and *SmRAS*) and terpenoid metabolic transcripts (*SmDXS*, *SmDXR*, and *SmHMGR*) [77,78]. Interestingly, overexpression of *SmbHLH148* not only upregulated the expression of tanshinone biosynthesis-related transcripts (e.g., *SmDXS*, *SmDXR*, and *SmHMGR*) but also induced the expression of SmJAZs involved in JA signaling pathways [78]. Additionally, SmWRKY1 and SmWRKY2 in *S. miltiorrhiza* significantly increase the transcription levels of the *SmDXS*, *SmDXR*, and *SmCPS* transcripts, thereby promoting terpenoid synthesis [79,80]. Furthermore, SmNAC1 in *S. miltiorrhiza* can activate *SmPAL* and *SmTAT* expression, leading to increased phenolic acid contents [81]. Similarly, the overexpression of *PfNAC29*, *PfNAC40*, and *PfNAC80* in *Perilla frutescens* hairy roots activated multiple key synthase transcripts in the phenylpropanoid pathway (e.g., *PfPAL*, *Pf4CL*, and *PfRAS*), significantly increasing the levels of phenolic acids such as RA, caffeic acid, and ferulic acid [82]. As *P. vulgaris*, *S. miltiorrhiza*, and *P. frutescens* are closely related Lamiaceae species, these results imply that SNP treatment is associated with changes in the expression of MYB, bHLH, WRKY, and NAC transcription factors, which may be involved in the regulation of polyphenol and triterpenoid biosynthesis in *P. vulgaris* seedling leaves; however, direct functional validation is required to confirm these relationships.

## 4. Materials and Methods

### 4.1. Plant Material and Treatment

Mature *P. vulgaris* seeds were collected in June 2023 from Shejian Town, Zhaohua District, Sichuan Province, China (latitude 32.25° N, longitude 105.43° E), with authentication performed by Professor Yuhang Chen from the College of Pharmacy, Chengdu Medical College. This experiment was carried out at the Medicinal Plant Garden of Chengdu Medical College, located at 30.82° N, 104.20° E, with an altitude of 445.4 m.

On 10 March 2024, the air-dried seeds were surface-sterilized to remove surface microorganisms: they were first immersed in 2% (*v*/*v*) H_2_O_2_ solution for 2 min with gentle shaking and then thoroughly rinsed three to four times with sterile distilled water (30 s per rinse). The sterilized seeds were subsequently soaked in purified water at 25 ± 1 °C for 12 h to promote imbibition and then evenly placed in sterile 90-mm diameter Petri dishes lined with two layers of moist sterile filter paper (100 seeds per dish) for germination. After 20 days, uniformly growing two-leaf-stage seedlings were selected and transferred to plastic seedling trays (50 holes per tray; individual hole dimensions: 5 × 5 cm in length/width, 10 cm in height). Each hole was filled with 25 ± 2 g of autoclaved substrate mixture (sandy loam:vermiculite = 20:1, *v*/*v*, pH = 7.23). The physicochemical properties of the substrate were measured in accordance with the relevant Chinese Agricultural and Forestry Industry Standards, and detailed parameters are presented in Supplementary Table S5.

After transplanting, the seedlings were regularly watered with sterile distilled water to maintain the relative soil water content at 60–70% of field capacity. All the seedlings were grown in an experimental greenhouse under the following ambient environmental conditions: 12/12 h light/dark photoperiod, day/night temperatures of 25 °C/10 °C, relative humidity ranging from 50% to 60% (monitored daily at 10:00 AM and 4:00 PM), and photosynthetically active radiation (PAR) levels of 700–1300 μmol m^−2^ s^−1^ (measured at midday with a PAR sensor, TES-1335, Taipei, Taiwan).

On 10 June 2024, approximately 70 days after transplanting, the exogenous SNP treatment was applied at 10:00 AM under sunny conditions. The ambient temperature was 25 ± 2 °C, and the PAR was 800 ± 50 μmol m^−2^ s^−1^. Sodium nitroprusside (SNP, AR grade, purity ≥ 99%, Macklin Biochemical Technology Co., Ltd., Shanghai, China) was used as the NO donor, and the 100 μM SNP aqueous solution was freshly prepared with sterile distilled water before treatment to avoid NO degradation. The seedlings were randomly divided into two groups with three independent biological replicates per group (six trays in total, 50 seedlings per tray, one plastic tray per biological replicate). Using an atomizing sprayer (nozzle diameter: 0.5 mm), each plastic seedling tray in the treatment group (SNP group) was uniformly sprayed with 200 mL of 100 μM SNP aqueous solution, while each plastic seedling tray in the control group (CK) was sprayed with 200 mL of sterile distilled water (the same volume as that in the treatment group). Spraying was continued until the droplets were nearly dripping from both leaf surfaces, ensuring full, uniform leaf coverage without causing mechanical damage to the seedlings.

Leaf samples were harvested at 2, 24, and 48 h after treatment to explore the responses of *P. vulgaris* to SNP treatment on physiological metabolism and transcript expression. For each time point, five seedlings were randomly selected from each replicate tray (15 seedlings per treatment per time point), and the apical pair of fully expanded true leaves was harvested from each plant. The leaves from the five seedlings per tray were pooled as one biological replicate. The collected fresh leaves were promptly frozen in liquid nitrogen for 5 min to terminate physiological reactions, wrapped in aluminum foil, and preserved at −80 °C for subsequent determination of physiological indicators, endogenous hormones, and transcriptomics. The 24 h time point (designated CK_24 vs. SNP_24) was selected specifically for transcriptomic profiling, as preliminary experiments revealed that the levels of key polyphenols and triterpenoids were the highest at this stage (Supplementary Figure S1).

Concurrently, an additional set of foliar samples was gathered at each time point to quantify the active ingredients and perform in vitro antioxidant activity assays. These samples were dried in a forced-air drying oven at 60 °C for 5 h until a constant weight was achieved, ground into fine powder, and sieved through a 60-mesh sterile sieve to ensure uniform particle size. All measurements were performed with three independent biological replicates to guarantee the reliability and reproducibility of the experimental data.

### 4.2. Physiological and Bioactive Component Analysis

#### 4.2.1. Determination of NO Levels, H_2_O_2_ Contents, Enzyme Activities, and Related Physiological and Biochemical Parameters

The activities of superoxide dismutase (SOD, EC 1.15.1.1), peroxidase (POD, EC 1.11.1.7), catalase (CAT, EC 1.11.1.6), ascorbate peroxidase (APX, EC 1.11.1.11), phenylalanine ammonia-lyase (PAL, EC 4.3.1.5), and polyphenol oxidase (PPO, EC 1.14.18.1), as well as the contents of malondialdehyde (MDA), free proline (Pro), soluble protein, soluble sugar, H_2_O_2_, NO, ascorbic acid (AsA), and glutathione (GSH), were measured using commercial assay kits. All detection kits were obtained from Nanjing Jiancheng Bioengineering Institute (Nanjing, China) and were used in accordance with the manufacturer’s protocols.

Specific detection wavelengths were used for each assay: 450 nm for SOD, 420 nm for POD, 405 nm for CAT, 290 nm for APX, 290 nm for PAL, 420 nm for PPO, 532 nm for MDA, 520 nm for Pro, 595 nm for soluble protein, 620 nm for soluble sugar, 405 nm for H_2_O_2_, 550 nm for NO, 536 nm for AsA, and 420 nm for GSH. Absorbance measurements were performed using a UV-5880 spectrophotometer (Unico(Shanghai) Instrumental Co., Shanghai, China).

All the results were calculated on the basis of the formulas specified in the respective kit instructions and are expressed as follows: SOD, POD, CAT, APX, and PAL activities in U mg^−1^ protein; PPO activity in U g^−1^ fresh weight (FW); MDA content in nmol g^−1^ protein; soluble protein content in g L^−1^; soluble sugar and Pro contents in μg g^−1^ FW; H_2_O_2_ content in mmol g^−1^ protein; NO content in μmol g^−1^ protein; AsA content in μg mg^−1^ protein; and GSH content in mg GSH g^−1^ protein.

#### 4.2.2. Determination of Endogenous Phytohormones

Ultrahigh-performance liquid chromatography–mass spectrometry (UHPLC–MS/MS) (ExionLC™ AD coupled with QTRAP^®^ 6500+, SCIEX, Framingham, MA, USA) was employed to determine the contents of endogenous hormones, namely, jasmonic acid (JA), salicylic acid (SA), and indole-3-acetic acid (IAA), in fresh *P. vulgaris* leaves.

Approximately 50.0 mg of frozen *P. vulgaris* leaves was ground in liquid nitrogen, followed by the addition of 1 mL precooled (4 °C) extractant (methanol/water/formic acid = 15:4:1, *v*/*v*/*v*) containing 10 μL of an internal standard and 0.1% BHT, thorough mixing and extraction by vortexing. The sample was then centrifuged at 4 °C and 12,000 rpm for 5 min. The resulting supernatant was concentrated using a vacuum concentrator, redissolved in 100 μL of 80% methanol, filtered through a 0.22-µm membrane, and transferred to a pre-cooled sample vial (4 °C) for UHPLC–MS/MS analysis.

Chromatographic separation was performed on a Waters ACQUITY UPLC HSS T3 C18 column (1.8 μm, 100 × 2.1 mm) at 40 ± 0.5 °C. The mobile phase consisted of A: ultrapure water (17.1 MΩ·cm) with 0.04% acetic acid, and B: acetonitrile with 0.04% acetic acid. The gradient program was as follows: 0–1.0 min, 5% B; 1.0–8.0 min, 5–95% B; 8.0–9.0 min, 95% B; 9.0–9.1 min, 95–5% B; 9.1–12.0 min, 5% B. The flow rate was 0.35 mL/min, the column temperature was 40 °C, and the injection volume was 2 μL.

Mass spectrometry was performed using a QTRAP^®^ 6500+ triple quadrupole linear ion trap mass spectrometer (Sciex) equipped with a Turbo V^TM^ electrospray ionization (ESI) source operating in positive/negative ion switching mode. The optimized MS parameters were as follows: curtain gas (nitrogen), 35 psi; nebulizer gas (Gas1), 55 psi; auxiliary heating gas (Gas2), 60 psi; positive ion spray voltage, +5500 V (with 0.1% formic acid optimization); negative ion spray voltage, −4500 V (with 5 mM ammonium acetate optimization); ion source temperature, 550 ± 2 °C. Multiple reaction monitoring (MRM) was used for quantification with a dwell time of 50 ms per channel and an inter-scan delay of 5 ms.

The MRM transitions and compound-dependent parameters (Q1, Q3, DP, CE) for IAA, JA, and SA are listed in Supplementary Table S6. The standard curve equations, correlation coefficients, linear ranges, and recovery are listed in Supplementary Table S7.

#### 4.2.3. Determination of Polyphenolic and Triterpenoid Contents

The content of total phenolic acids was quantified using a modified Folin–Ciocalteu assay [16]. Briefly, 0.5 g of dried leaf powder was subjected to ultrasonic extraction with 50 mL of 70% ethanol for 60 min. After filtration, the extract was mixed with Folin–Ciocalteu reagent and sodium carbonate, and the absorbance was measured at 760 nm. Quantification was performed using a gallic acid standard curve (0.01–0.10 mg mL^−1^, R^2^ > 0.999).

Total triterpenoids were quantified via a colorimetric method [83]. Dried powder (0.5 g) was ultrasonically extracted with 50 mL of methanol, and the extract was reacted with vanillin-acetic acid and perchloric acid. The absorbance of the colored complex was measured at 545 nm, with oleanolic acid serving as the standard (0.0148–0.0888 mg mL^−1^, R^2^ > 0.999).

The total flavonoid content was measured following the method described previously [16]: 0.5 g of powdered sample was ultrasonically extracted with 20 mL of 50% ethanol, and the filtrate was mixed with sodium nitrite, aluminum nitrate, and sodium hydroxide. After 10 min, the absorbance was recorded at 510 nm using a rutin standard curve (0–1.6 mg mL^−1^, R^2^ > 0.999).

The contents of RA and UA were analyzed via HPLC with diode-array detection [16]. For sample preparation, RA was extracted from 0.2 g of powder by ultrasonic treatment with 15 mL of 80% methanol at 50 °C twice, and the combined extracts were concentrated and filtered; UA was obtained by ultrasonic extraction of 1.0 g of powder with 10 mL of methanol, followed by centrifugation and filtration.

HPLC conditions: Analyses were performed on an Elite EClassical 3200 HPLC system fitted with a Chrom Plus C18 column (250 mm × 4.6 mm, 5 μm). RA was detected at 325 nm using gradient elution with acetonitrile and 0.1% phosphoric acid (flow rate 0.8 mL min^−1^, column temperature 30 °C). UA was detected at 205 nm via isocratic elution with methanol and 0.2% acetic acid (90:10, *v*/*v*) at a flow rate of 0.8 mL min^−1^ and a column temperature of 18 °C. The injection volume was 10 μL for both analyses.

#### 4.2.4. In Vitro Antioxidant Activity Assay and Correlation Analysis

The in vitro antioxidant activity of *P. vulgaris* leaf extracts was evaluated by determining their scavenging capacities against DPPH and ABTS radicals.

For sample preparation, 1.00 g of dried leaf powder was ultrasonically extracted with 30 mL of 70% ethanol at 70 °C for 30 min. After filtration, the residue was re-extracted twice under identical conditions, and the combined filtrates were concentrated and diluted with absolute ethanol to prepare serial working solutions for subsequent assays.

The DPPH radical scavenging capacity was assessed according to the method reported previously [2]. In brief, 1.0 mL of sample solutions with varying concentrations was mixed with 4.0 mL of freshly prepared DPPH-methanol solution (0.04 mg mL^−1^). Following 30 min of incubation in the dark at room temperature, the absorbance was measured at 517 nm. Scavenging activity was calculated, and the half-maximal inhibitory concentration (IC_50_) was determined via a four-parameter logistic (4PL) regression model.

The ABTS radical scavenging capacity was determined following the protocol described previously [84]. The ABTS radical cation working solution was prepared by oxidizing 7 mmol L^−1^ ABTS with 2.45 mmol L^−1^ potassium persulfate in the dark for 16 h and then diluting to an absorbance of 0.70 ± 0.02 at 734 nm. A 1.0 mL sample solution was mixed with 1.0 mL of the ABTS working solution; after 6 min of incubation in the dark, the absorbance was measured at 734 nm. The IC_50_ values were calculated as described for the DPPH assay. For the data at different time points (2, 24, and 48 h), significant differences among treatments at each time point were determined by one-way analysis of variance (ANOVA) with LSD post hoc comparisons (*p* < 0.05).

### 4.3. P. vulgaris Transcriptome Analysis

#### 4.3.1. Total RNA Sequencing

TRIzol^®^ reagent (Invitrogen, Carlsbad, CA, USA) was used to extract total RNA from *P. vulgaris* leaf samples. RNA quality and integrity were verified using a Nanodrop spectrophotometer (A260/280 = 1.8–2.1), an Agilent 2100 Bioanalyzer (RIN ≥ 8.0, DV200 > 80%), and a Qubit fluorometer for RNA concentration measurement. Oligo (dT) magnetic beads from the NEBNext^®^ Poly(A) mRNA Magnetic Isolation Module were employed to enrich poly(A)+ mRNA. Full-length cDNA was generated using the SMARTer^®^ PCR cDNA Synthesis Kit, optimized by gradient PCR (annealing temperature: 58–68 °C; cycles: 12–18), and size-selected (0.5–6 kb) with AMPure XP beads. After cDNA repair, end polishing and dA tailing, circular libraries were constructed using the SMRTbell^®^ Express Template Prep Kit 2.0, and Exo VII was used to eliminate unclosed linear molecules. Qualified libraries (concentration ≥ 30 ng·μL^−1^, fragment size 1–10 kb) were sequenced on a PacBio Sequel IIe system (CCS mode, ≥3 passes) and an Illumina NovaSeq 6000 platform (PE150 mode) for data correction and transcript reconstruction using the LoRDEC algorithm. Detailed RNA-seq quality metrics, including sequencing depth, Q20/Q30, GC content, mapping rates, and biological replicate correlations, are summarized in Supplementary Table S8.

#### 4.3.2. Full-Length Transcriptome Analysis

PacBio raw polymerase reads were processed with SMRT Link v11.0 to produce subreads (N50 > 15 kb), followed by CCS iteration correction (Passes ≥ 3, predicted accuracy ≥ 99%). Full-length nonchimeric (FLNC) sequences were screened using Iso-Seq3.4.0 (concatemer rate < 0.1%) and clustered with the isoseq3 cluster (min-alignment coverage 0.85) to generate consensus transcripts (Polish accuracy > Q30). Statistics for CCS and FLNC reads are provided in Supplementary Table S2. Redundant transcripts were removed by CD-HIT v4.8.1 (identity = 0.95, coverage = 0.90) to construct a high-confidence reference transcriptome. These nonredundant transcripts, which may contain merged isoforms/paralogs, were used as the reference for quantification and are referred to as transcripts rather than genes or individual isoforms unless unambiguously assigned to a transcript locus. Illumina short reads were aligned to this reference using Bowtie2 v2.4.5, and transcript expression levels were determined with RSEM v1.3.3. Raw counts were used for differential expression analysis, while FPKM values were calculated only for heatmap visualization.

#### 4.3.3. Transcript Annotation and Differential Expression

Nonredundant transcripts were functionally annotated against NCBI NR 2024, UniProt/Swiss-Prot 2024-Q2, KEGG 2023, COG/KOG (EggNOG 5.0), and Pfam (InterProScan 92.0) using DIAMOND v2.1.8 and HMMER3.3.2. Differential transcript expression analysis between the treatment and control groups was performed using DESeq2 v1.40.2 based on raw counts, with DETs identified by FDR < 0.01 (Benjamini–Hochberg FDR correction) and |log_2_-fold change| ≥ 1. GO and KEGG pathway enrichment analyses were carried out on the DETs to explore pathways related to antioxidant defense, endogenous hormones, and bioactive component biosynthesis. Transcription factors (TFs) were predicted using iTAK (v1.7) software.

#### 4.3.4. RT-qPCR Validation

Twenty-one DETs were chosen for RT-qPCR verification, with β-actin serving as the internal reference transcript [16]. Transcript-specific primers were designed using Primer Premier 5 (Supplementary Table S9). Total RNA (500 ng) was first treated to remove gDNA and then reverse-transcribed into cDNA using HiScript II qRT SuperMix in a 20 μL reaction system. RT-qPCR was conducted in 20 μL volumes, containing 4 μL of diluted cDNA, 0.4 μM of each primer, 10 μL of SYBR Green Master Mix, 0.4 μL of 50×ROX Reference Dye 2, and 4.8 μL of H_2_O, on a real-time PCR instrument. The reaction program included initial denaturation at 95 °C for 10 min, followed by 40 cycles of 95 °C for 15 s and 60 °C for 60 s, and a subsequent melting curve analysis. Relative transcript expression levels were computed using the 2^−ΔΔCt^ method, with three biological replicates for each sample.

### 4.4. Statistical Analysis

All the quantitative data are expressed as the mean ± standard deviation (SD) from three independent biological replicates. Statistical analyses were carried out with SPSS Statistics software 21.0 (IBM Corp., Armonk, NY, USA). Significant differences among treatment groups were determined by one-way ANOVA combined with the LSD method for post hoc tests, with significant differences set at *p* < 0.05. GraphPad Prism 8.0.2 was used for data visualization and curve fitting (e.g., for the IC_50_ calculations). DET expression heatmaps were created using GraphPad Prism 8.0.

## 5. Conclusions

Overall, the integrated physiological and transcriptomic analyses revealed that exogenous SNP treatment induced physiological responses in *P. vulgaris* seedling leaves over the 2–48 h treatment period. First, SNP significantly altered antioxidant and redox responses, elevated the content of osmotic regulators, and promoted the accumulation of polyphenolic and triterpenoid compounds (e.g., RA and UA), accompanied by increased in vitro antioxidant capacity. Second, at 24 h post treatment, 4341 DETs were identified between the CK and SNP treatment groups. SNP treatment was associated with changes in phytohormone signal transduction, Ca^2+^ signaling, and secondary metabolite biosynthesis. Third, SNP treatment markedly increased endogenous JA levels and upregulated the expression of core transcription factors (MYBs, bHLHs, WRKYs, and NACs), along with JA biosynthetic transcripts and key structural transcripts involved in polyphenol and triterpenoid biosynthesis. Building on these findings, future research will focus on the molecular processes of additional stress-related signaling molecules, such as H_2_O_2_ and Ca^2+^, which may act synergistically or in concert with SNP-induced signaling to fine-tune secondary metabolism. Moreover, we aim to elucidate the upstream regulatory cascades involving key TFs (e.g., MYBs, bHLHs, WRKYs, and NACs) and to explore the potential roles of small non-coding RNAs (miRNAs) in mediating the posttranscriptional regulation of polyphenol and triterpenoid biosynthesis under various abiotic stress conditions. This study provides valuable insights into the physiological and transcriptional responses through which exogenous SNP modulates secondary metabolite production in *P. vulgaris* seedling leaves, and may provide a theoretical foundation for future field studies on SNP application and understory ecological cultivation to improve the quality of this valuable edible-medicinal plant.

## Data Availability

The raw sequencing data have been deposited in the Genome Sequence Archive (GSA) under accession number CRA051030.

## Supplementary Materials

The following supporting information can be downloaded at https://www.mdpi.com/article/10.3390/ijms27219585/s1.
