# Supplementary material for: Integrated Physiological and Transcriptomic Analyses Reveal Secondary Metabolic Responses of Prunella vulgaris Seedlings to Sodium Nitroprusside Treatment
Source: Int J Mol Sci. 2026 Oct 27;27(21):9585. doi: 10.3390/ijms27219585 (PMC13763079; doi:10.3390/ijms27219585)
Supplement: Supplementary file 1 [file ijms-27-09585-s001.zip › ijms-4556331-supplementary.pdf]

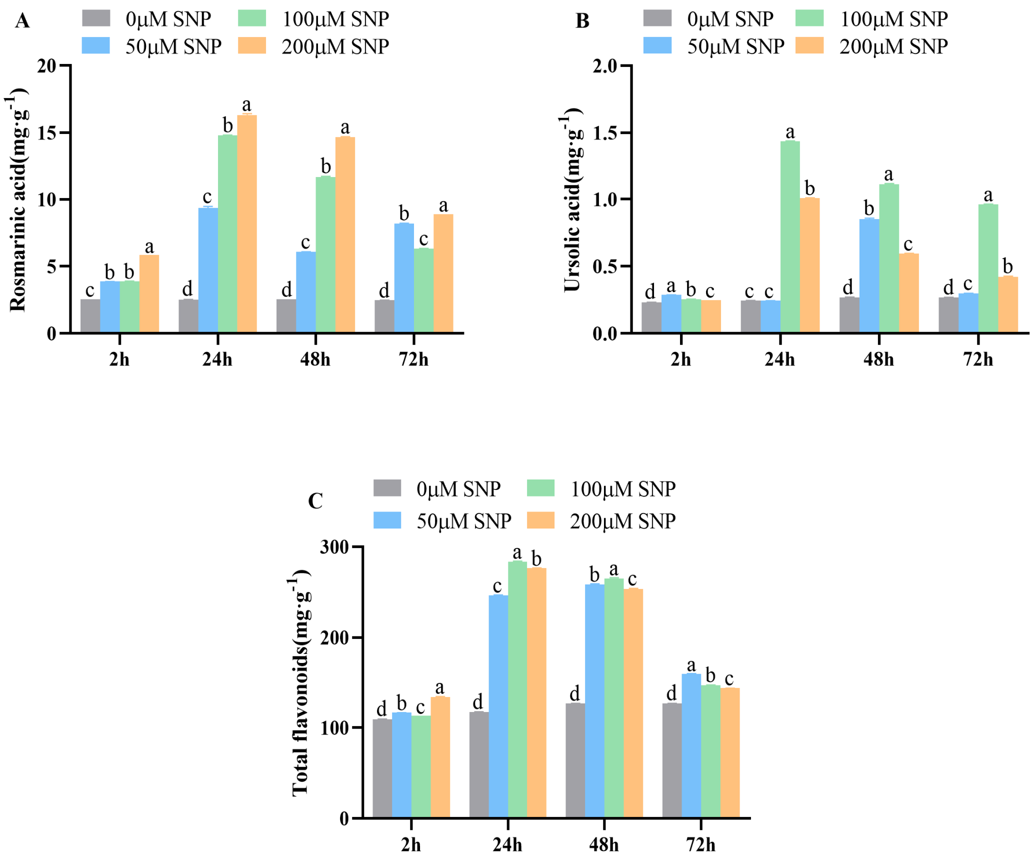


**Supplementary Figure S1.** Influence of different exogenous SNP concentrations on key bioactive compound levels in *P. vulgaris* seedling leaves over 72 h. (A) Rosmarinic acid (RA) level. (B) Ursolic acid (UA) level. (C) Total flavonoid level. The seedlings were treated with 0, 50, 100, or 200 μM SNP, and the samples were harvested at 2, 24, 48, and 72 h after treatment. The data are expressed as the mean ± SD (n = 3). Different lowercase letters denote significant differences between treatments at the same time point ($p < 0.05$, one-way ANOVA followed by the LSD test).

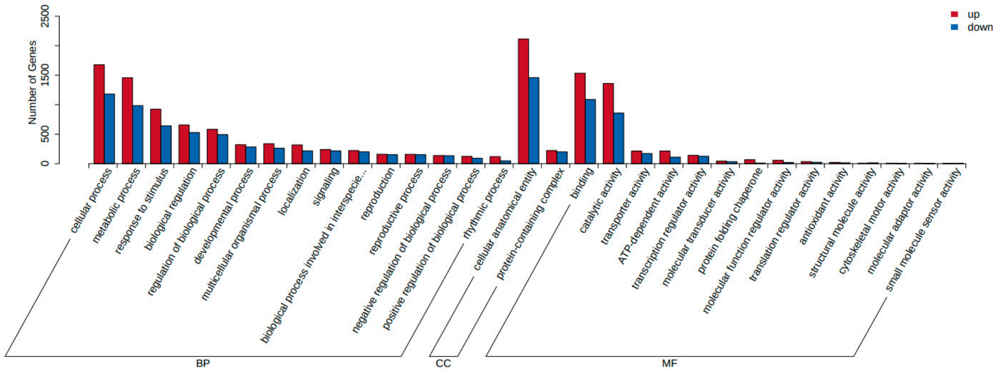


**Supplementary Figure S2.** Gene Ontology (GO) functional classification of DEGs identified between the control (CK) and 100 μM SNP-treated *P. vulgaris* leaves at 24 h post treatment.

**Supplementary Table S1.**

Statistics of polymerase reads from PacBio Iso-Seq sequencing of *P. vulgaris* leaf transcriptome.

| Sample | Polymerase read bases (Gb) | Polymerase reads | Polymerase read average length (bp) | Polymerase read N50 (bp) |
|---|---|---|---|---|
| *P. vulgaris* leaves | 50.45 | 353,897 | 142,563 | 210,376 |

**Supplementary Table S2.**

Statistics of CCS and FLNC readsfrom PacBio Iso-Seq sequencing of *P. vulgaris* leaf transcriptome.

| Sample | CCS_bases (bp) | CCS_number | Min_length (bp) | Average_length (bp) | Max_length (bp) | N50 (bp) |
|---|---|---|---|---|---|---|
| *P. vulgaris* leaves | 669,375,822 | 298,991 | 93 | 2,239 | 8,485 | 2,533 |
| Sample | FLNC_bases (bp) | FLNC_number | Min_length (bp) | Average_length (bp) | Max_length (bp) | N50 (bp) |
| *P. vulgaris* leaves | 603,847,237 | 280,786 | 57 | 2,151 | 8,408 | 2,459 |

**Supplementary Table S3.**

Transcript length-frequency distribution and summary statistics before and after deredundancy.

| Sample | Type | <500 bp | 500 bp-1 kbp | 1-2 kbp | 2-3 kbp | >3 kbp | Total |
|---|---|---|---|---|---|---|---|
| *P. vulgaris* leaves | Before de-redundancy | 4 | 1266 | 11,122 | 11,516 | 6252 | 30,160 |
| | After de-redundancy | 4 | 1095 | 9035 | 9319 | 5183 | 24,636 |
| Summary statistics | | | | | | | |
| Type | Total nucleotides | Total number | Mean length (bp) | Min length (bp) | Max length (bp) | N50 (bp) | N90 (bp) |
| Before de-redundancy | 66,722,249 | 30,160 | 2213 | 301 | 7161 | 2480 | 1434 |
| After de-redundancy | 66,699,564 | 30,160 | 2212 | 301 | 7161 | 2478 | 1433 |

**Supplementary Table S4.**

Count of up- and downregulated DEGs in key KEGG pathways associated with polyphenol metabolism, terpenoid metabolism, plant hormone signal transduction, and plant–pathogen interaction (CK vs. SNP at 24 h).

| CK vs. SNP | KEGG pathway | Genes | Number |
|---|---|---|---|
| Up DEGs | Phenylpropanoid biosynthesis | *PvPAL*、*PvC4H*、*Pv4CL* | 41 |
| | Phenylalanine metabolism | *PvPAL*、*PvTAT*、*PvPAL* | 24 |
| | Diterpenoid biosynthesis | *PvTPS* | 5 |
| | Terpenoid backbone biosynthesis | *PvDXS*、*PvHMGS* | 32 |
| | Monoterpenoid biosynthesis | *Hypothetical protein* | 1 |
| | Sesquiterpenoid and triterpenoid biosynthesis | *PvOSC*、*PvSQE* | 7 |
| | Plant hormone signal transduction | *PvJAZ*、*PvMYC2*、*PvACX* | 47 |
| | Plant-pathogen interaction | *PvCDPK*、*PvCNGC* | 129 |
| Down DEGs | Phenylpropanoid biosynthesis | *PAL-like* | 1 |
| | Phenylalanine metabolism | *PAL-like* | 2 |
| | Diterpenoid biosynthesis | *Hypothetical protein* | 1 |
| | Terpenoid backbone biosynthesis | *PvHMGR* | 11 |
| | Monoterpenoid biosynthesis | *Geraniol dehydrogenase 1* | 2 |
| | Sesquiterpenoid and triterpenoid biosynthesis | *Pvβ-AS* | 6 |
| | Plant hormone signal transduction | *PvCOI1*、*PvTAA1*、*PvYUCCA* | 78 |
| | Plant-pathogen interaction | *PvbHLH* | 115 |

**Supplementary Table S5.**

Physicochemical characteristics of the substrate used in the seedling trays.

| Parameter | Standard method | Measured value |
|---|---|---|
| Organic matter (g $kg^{-1}$) | NY/T 1121.6-2006 | 818.33 |
| Available phosphorus (g $kg^{-1}$) | NY/T 1121.7-2014 | 0.088 |
| Available potassium (g $kg^{-1}$) | NY/T 889-2004 | 0.078 |
| Hydrolyzable nitrogen (g $kg^{-1}$) | LY/T 1228-2015 | 0.344 |

Note: NY / T: Recommended agricultural industry standard of China; LY / T: Recommended forestry industry standard of China.

**Supplementary Table S6.**

MRM transitions and compound-dependent parameters for the target hormones.

| Compound | Q1 (m/z) | Q3 (m/z) | DP (V) | CE (V) |
|---|---|---|---|---|
| IAA | 176.1 $[M+H]^+$ | 130.1 | 20 | 20 |
| JA | 209.1 $[M-H]^-$ | 58.8 | −6 | −5 |
| SA | 137.0 $[M-H]^-$ | 93.0 | −20 | −20 |

**Supplementary Table S7.**

Calibration parameters for the target hormones.

| Compound | Linear range (ng/mL) | Equation | r | LLOQ (ng/mL) | ULOQ (ng/mL) | RSD (%) | Recovery (%) |
|---|---|---|---|---|---|---|---|
| IAA | 0.01–500 | y = 0.01179 x + 2.73943e-5 | 0.99947 | 0.2 | 500 | 5.73 | 91.92 |
| JA | 0.1–500 | y = 0.05932 x + 0.00225 | 0.99791 | 0.2 | 500 | 7.51 | 102.21 |
| SA | 0.5–500 | y = 0.07851 x + 0.00701 | 0.99425 | 0.5 | 500 | 7.08 | 80.58 |

**Supplementary Table S8.**

RNA-seq sequencing depth, quality, mapping, and correlation statistics.

| Sample | Raw Reads | Clean Base (G) | Q20 (%) | Q30 (%) | GC (%) | Mapping Rate | Correlation Range |
|---|---|---|---|---|---|---|---|
| CK-1 | 62,512,184 | 9.21 | 97.25 | 92.25 | 47.79 | 66.82% | 0.83 - 0.98 |
| CK-2 | 53,261,370 | 7.80 | 97.34 | 92.52 | 47.36 | 65.43% | 0.71 - 0.83 |
| CK-3 | 57,303,098 | 8.44 | 97.19 | 92.08 | 48.08 | 67.86% | 0.71 - 0.98 |
| SNP-1 | 54,787,668 | 8.06 | 97.25 | 92.27 | 47.52 | 67.76% | 0.97 - 0.99 |
| SNP-2 | 57,076,812 | 8.39 | 97.24 | 92.20 | 47.62 | 68.36% | 0.93 - 0.97 |
| SNP-3 | 54,706,494 | 8.04 | 97.15 | 92.00 | 47.56 | 68.35% | 0.93 - 0.99 |

Note: The intra-group correlation range represents the Pearson correlation coefficients among the three biological replicates within each group.

**Supplementary Table S9.**

Primer sequences employed for RT–qPCR detection.

| No | Gene name | Forward primer (5'–3') | Reverse primer (5'–3') |
|---|---|---|---|
| 1 | *β-actin* | GACCAGCTCTGCTGTGGAGA | ATGGCTGGAAGAGGACCTCAG |
| 2 | *PvPAL* | AGGAAGACCGTGGAGGC | ATGGTGAGGGTGCGTTT |
| 3 | *PvC4H* | ATGGTCGATTGAGTGGG | CCTGAAGGTATGGGAGC |
| 4 | *PvTAT* | GCTCGCCCTGGCTGTAA | ATCAACCTCCCATCCCT |
| 5 | *PvCYP98A* | TCGCACTCTTCTCCGCTAA | AGGCACCAATCCTCAATCTG |

| | | | |
|---|---|---|---|
| 6 | *Pv4CL* | CTGAAGGCAAGAAGAGTAGGT | AAGGAGGGTGTATGTATAATGTG |
| 7 | *PvRAS* | CGATAGGGAGGCTGGAC | CGGGATTGGGAAGAAGA |
| 8 | *PvHMGS* | CAGTTCTCGGTCTTGGA | GCACCTTGGCATCATAG |
| 9 | *PvHMGR* | GCCATCAGAAGAAAGTCG | GTAAACAACGGAGAAGAACA |
| 10 | *PvSQS* | TGTTGGACTAGGGTTGT | ATCCGTGACTTTGGTAT |
| 11 | *PvSQE* | CACTCGGCTTTCCTACC | ACGGGACATCCACTTTT |
| 12 | *PvDXS* | TCCACATCATCACCGAGAA | GGCGAAGTATTGCGTGT |
| 13 | *PvHDS* | GGTGACGAACCCGAAGA | GGTCCCAGCACCGATAA |
| 14 | *PvHDR* | TGTGGTCTTACCTGCTT | CTTACCGTGGATTACTGA |
| 15 | *PvAOC* | GCACAAGCGGTTGAATA | TTGTTGGAGAAAGGCAC |
| 16 | *PvCOI1* | CTTGGGACTTACTTACCTG | GCTCCACGCTCTATTCT |
| 17 | *PvJAZ* | AAGGTGCTACCGACAAA | CAGTCTCAGGTGCCAAA |
| 18 | *bHLH* | AATGTGGCAAGAAGAACC | GAATCCAGTCACCGAGTT |
| 19 | *bHLH* | TTATGAATGCGTTCCCAGTT | GGTGATGGTGGCTTGGTC |
| 20 | *MYB-related* | CGCATCATCTCGCTTGT | TTTCTTGCTGTCGTCCA |
| 21 | *PvCDPK* | CCTTGTCCGAAGACGAAA | GGTGATGTAGCCACTCCC |
| 22 | *PvCNGC2* | GAAGCAAGGCATCTCAG | GTTCACAGCAGCCCAAG |
